# The effects of anthropomorphism and multimodal biometric authentication on the user experience of voice intelligence

**DOI:** 10.3389/frai.2022.831046

**Published:** 2022-08-17

**Authors:** Mels de Kloet, Shengyun Yang

**Affiliations:** Research Centre Business Innovation, Rotterdam University of Applied Sciences, Rotterdam, Netherlands

**Keywords:** anthropomorphism, artificial intelligence, customer value chain, multimodal biometric authentication, user perception, voice intelligence

## Abstract

Voice intelligence is a revolutionary “zero-touch” type of human-machine interaction based on spoken language. There has been a recent increase in the number and variations of voice assistants and applications that help users to acquire information. The increased popularity of voice intelligence, however, has not been reflected in the customer value chain. Current research on the socio-technological aspects of human-technology interaction has emphasized the importance of anthropomorphism and user identification in the adoption of the technology. Prior research has also pointed out that user perception toward the technology is key to its adoption. Therefore, this research examines how anthropomorphism and multimodal biometric authentication influence the adoption of voice intelligence through user perception in the customer value chain. In this study we conducted a between-subjects online experiment. We designed a 2 × 2 factorial experiment by manipulating anthropomorphism and multimodal biometric authentication into four conditions, namely *with* and *without* a combination of these two factors. Subjects were recruited from Amazon MTurk platform and randomly assigned to one of the four conditions. The results drawn from the empirical study showed a significant direct positive effect of anthropomorphism and multimodal biometric authentication on user adoption of voice intelligence in the customer value chain. Moreover, the effect of anthropomorphism is partially mediated by users' perceived ease of use, perceived usefulness, and perceived security risk. This research contributes to the existing literature on human-computer interaction and voice intelligence by empirically testing the simultaneous impact of anthropomorphism and biometric authentication on users' experience of the technology. The study also provides practitioners who wish to adopt voice intelligence in the commercial environment with insights into the user interface design.

## Introduction

The rapid technological development of artificial intelligence (AI), particularly machine learning and Natural Language Processing (NLP), have made it possible to transform the binary language of computers into understandable verbalized output (Hirschberg and Manning, 2015) and have enabled the voice user interface, also called “voice intelligence”. Voice intelligence accepts voice as input, after which it is processed and transformed into voice-based output (Oberoi, 2019) and responds with answers similar to everyday real-time human-to-human interaction. In turn, it has led to a “zero-touch” user interface, demonstrating how human-machine interaction has shifted away from screens and keyboards (Pemberton, 2018) with the use of biometrics of the human body (in this case, voice) in this interaction process (Mahfouz et al., 2017).

Voice intelligence is seen as the third key user interface of the past three decades, following the World Wide Web and smartphones (Kinsella, 2019). Both the World Wide Web and smartphones require users to learn new languages and interaction methods before they can successfully use the interfaces (Kinsella, 2019). However, voice intelligence differs from its predecessors in that it does not engender any learning curve among its users but accepts their voices as a natural interface. As such, it has been predicted that there will be a growing interest in voice intelligence (Kinsella, 2020).

In addition to offering an improved user experience, voice intelligence has become a solution for the sub-optimal connectivity of a previously neglected segment of the world's population (Chérif and Lemoine, 2019; ITU, 2019; Lee and Yang, 2019). First, for individuals who are unable to fully interact due to illiteracy, physical or motorial disability, arthritis, or reading impairments (e.g., dyslexia), voice intelligence allows them to more fully access the Internet (Chérif and Lemoine, 2019; Max Planck Institute for Psycholinguistics, 2019). Second, in many developing countries, the issue of written language inconsistencies are pervasive, such as a lack of a standard keyboard alphabet. Meanwhile, over 75% of the developing world's population actively uses a mobile broadband subscription. Therefore, voice intelligence can be a crucial and leveling intermediary for populations in developing countries to access the Internet, information, and online services. In short, voice intelligence makes a practical contribution to inclusiveness and helps remove barriers to digital accessibility worldwide (Delić et al., 2014).

Meanwhile, voice intelligence can create new business opportunities by being an intermediary in human-computer interaction (HCI), connecting users with results applicable to their needs (Liu, 2021). Along with the growing “always-online” mentality (Chuah et al., 2016; Rauschnabel et al., 2018) and the increased number of recognized languages due to good development trends, it has been estimated that voice intelligence will have a market value of USD 7.7 billion and a target population surpassing one billion recurring users by 2025 (Kinsella, 2019). This remarkable potential implies that as consumers' first-choice of platforms, voice intelligence software will regulate a substantial part of the customer value chain (Kinsella, 2019). Leading big tech companies recognize this transition and are thus challenging each other to become market leaders (Liu, 2021).

Nevertheless, consumer adoption of voice intelligence remains underdeveloped (PWC, 2018). Multiple technological and social characteristics form barriers. For example, privacy concerns, security risks, social acceptability, and user hesitancy toward the technological capabilities are identified as fundamental problems decelerating the consumer adoption progress (Moorthy and Vu, 2015; Efthymiou and Halvey, 2016; Bajorek, 2019; Zhang et al., 2019).

Furthermore, most current voice intelligence users are “early adopters” (Moore, 1991; Kinsella, 2019). Early adopters are characterized by their lower loyalty toward technologies due to the excitement they experience from trying out new products (Moore, 1991). Stepping up the adoption curve is crucial for continuing the development of voice intelligence (Rogers, 1995). Therefore, further research is needed to overcome prior adoption barriers and to guide the transformation process.

This study investigates, from a socio-technological perspective, the different characteristics and features of voice intelligence that need to be transformed in order to raise user perception of it to a level equivalent to that of stationary computers or mobile phones. This acknowledgment is essential for establishing a sustainable human-technology relationship that meets the product standards of current technological intermediaries (Moore, 1991).

In this research, we propose that incorporating anthropomorphism and multimodal biometric authentication enhances users' perception toward voice intelligence, improving their experiences of the relational exchanges and thus increasing their willingness to adopt voice intelligence in the customer value chain. Accordingly, the following research question was put forth:

*How do anthropomorphism and multimodal biometric authentication influence the adoption of voice intelligence in becoming an acknowledged intermediate technology in the customer value chain?*

To empirically validate the hypotheses, we conducted a 2 × 2 online experiment. We designed and developed four conditions based on with or without anthropomorphic characteristics and multimodal biometric authentication in collaboration with the Amazon developer community. Two hundred and forty subjects were recruited *via* Amazon Mechanical Turk (MTurk), a crowdsourcing platform.

The results confirm the influence of anthropomorphic characteristics and multimodal biometric authentication on user experience in the customer value chain, and thus further lead to an impact on the adoption of voice intelligence. While the former predictor has an impact on specific user perceptions, the latter influences the aggregate level of user perception.

The structure of this paper is as follows: Section Theory and hypothesis development presents the theoretical background of this study and hypothesis development. Section Research method delineates the research methodology, including the experiment design and measures of variables. Section Analysis and results elaborates on the results drawn from the experiment and validates the hypotheses. Section Discussion further discusses the results. Section Conclusion concludes with a summary of the findings and academic as well as practical contributions.

## Theory and hypothesis development

**Figure 1:**
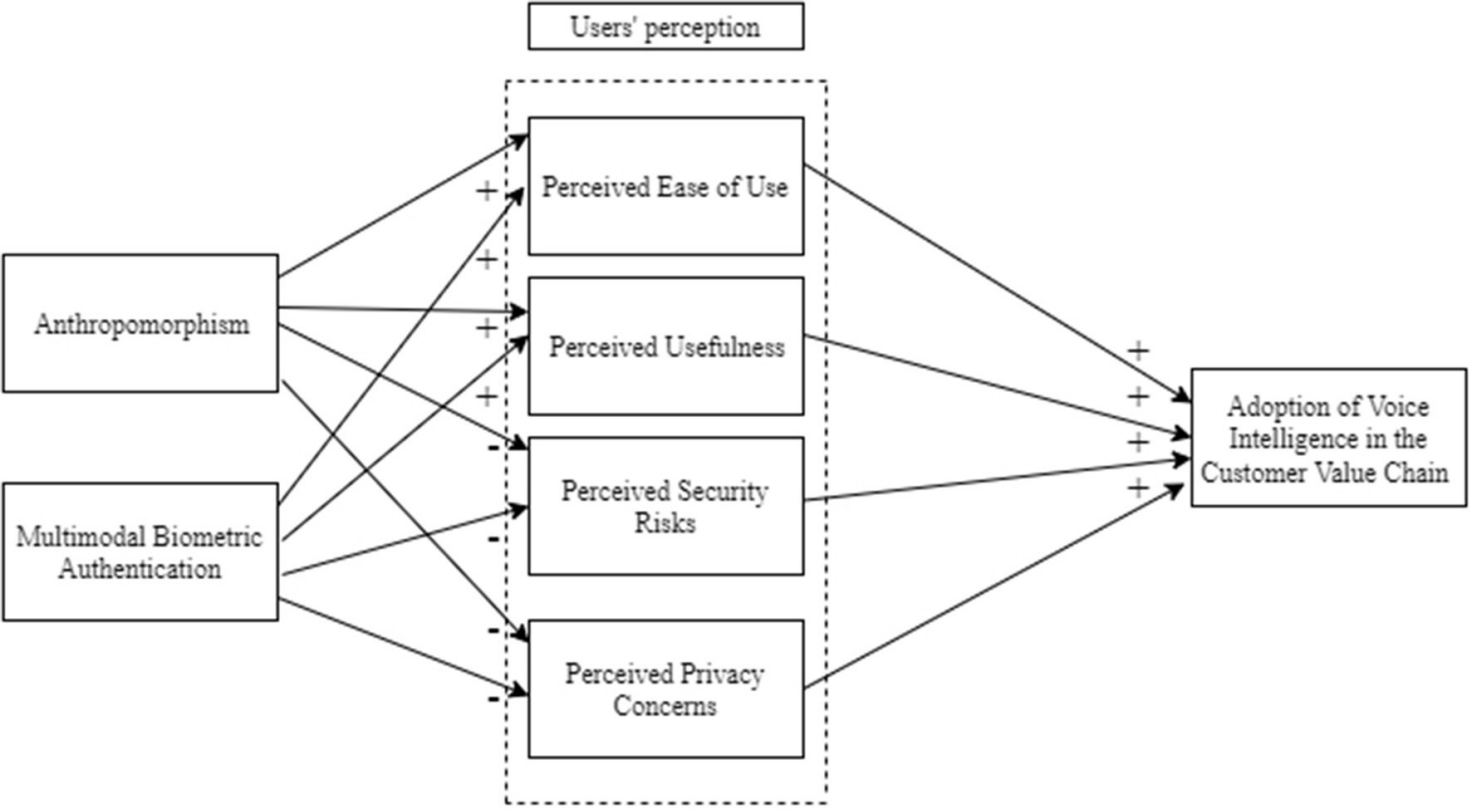
Research model.

In this section, we review the literature on the voice intelligence, human-technology interaction, and user behavior in the digital age. Thereafter, we present the research model and develop our hypotheses based on the literature review. Figure 1 illustrates the research model and hypothesized association between the variables.

### Voice intelligence

Voice intelligence, based on AI, has realized a long-existing human desire to communicate with technology through a natural interface (Hoy, 2018). The unique linguistic software of voice intelligence forms the base that makes it possible to establish a successful dialogue between humans and technology. Nevertheless, customer awareness and adoption of voice intelligence are nascent and thus underdeveloped (PWC, 2018). As such, we commenced our literature survey with voice intelligence to better understand its fundamentals. This subsection introduces the voice intelligence process, its current applications in different domains, and its advantages and challenges.

#### The voice intelligence process

The unique linguistic software of voice intelligence forms the base for establishing a successful dialogue. Conversational agents in the form of chatbots or virtual assistants are interfaces that apply voice intelligence to communicate with users (Janssen et al., 2020). After hearing specific keywords, such as “Hey Alexa” or “Ok Google”, voice software within virtual assistants and chatbots redirects the user input to a specialized server. Subsequently, the server transforms the information into a command. This command then triggers voice intelligence to respond by providing the requested information, activating an application, executing a given task independently, or interacting with connected devices (Amazon, n.d.). The virtual assistants called Alexa or Google assistant are classified as intelligent because of their ability to apply cloud-based text-to-speech and speech-to-text services as initial reaction on the given user input (Jadczyk et al., 2021). Accordingly, virtual assistants or chatbots are conversational software tools that use artificial voice intelligence and NLP-technology to support users in their daily lives (Janssen et al., 2020).

During the voice intelligence process, four essential elements within voice intelligence cooperate consecutively with each other to successfully effectuate the intended user experience and output (de Barcelos Silva et al., 2020). Table 1 explains the different voice intelligence components and how these elements successively work together.

**Table 1:** Components of voice intelligence (Pieraccini, 2012; Hirschberg and Manning, 2015; de Barcelos Silva et al., 2020; Amazon, 2021).

**Components**	**Functions**
Automated Speech Recognition (ASR)	Coverts the given input into understandable commands by matching words with patterns in sound peaks.
Natural Language Processing (NLP)	Semantically structures the linguistic utterance using computational techniques.
Dialog Management (DM)	Decides which action should be performed according to previous interactions' dialog strategy and experiences.
Response Generator (RG)	Produces the output text and synthesizes it to voice.

#### Applications of the voice intelligence process

The current applications of voice intelligence can be classified into non-commercial and commercial uses. Non-commercial applications are commonly seen in the educational and medical environments (de Barcelos Silva et al., 2020). Voice intelligence is primarily used in educational settings to learn a second language or for student assistance purposes (Zhu et al., 2007; Todorov et al., 2018). NLP makes it beneficial to use voice intelligence software for learning foreign languages due to its textual aspect and familiar way of interacting (Zhu et al., 2014). This personal feeling enables users to comfortably interact with a system that records their voice for review purposes (Todorov et al., 2018). This personalized relationship helps to improve recognizability and the user experience by learning from user linguistic data (Zhu et al., 2014). Based on this extensive user profile, voice intelligence software can further tailor teaching methods to the capabilities of their users (Todorov et al., 2018).

The implications of voice intelligence in healthcare are vast, varying from taking over mandatory data collection tasks to improving healthcare's accessibility (de Barcelos Silva et al., 2020). The COVID-19 pandemic started a re-evaluation of the doctor-patient relationship (Pederson and Jalaliniya, 2015). The abrupt increase in healthcare intensity exposed the vulnerability of relying almost exclusively on a face-to-face care delivery model (Sezgin et al., 2020; McKinsey and Company, 2021). Accordingly, during the pandemic the healthcare industry in the United States utilized voice intelligence in various initiatives, varying from information distribution to question handling (de Barcelos Silva et al., 2020). This continuous remote healthcare management and the role of voice intelligence as a supportive application was able to provide a solution for many vulnerable population groups (Sezgin et al., 2020).

The use of voice intelligence in the commercial environment is demonstrated in the customer value chain. The customer value chain consists of three phases: evaluation, purchase, and use (Cuofano, 2021). The users' focus in this value chain is to find products or services that correctly serve their needs (Lazarus, 2001). Voice intelligence can play a vital role in the evaluation and purchase phases by improving the friction–time trade-off, which explains how perceived convenience affects the customer experience (Kemp, 2019). Voice intelligence improves this trade-off by providing less friction and saving valuable time during the interaction (Guzman, 2018; Kemp, 2019). The time it takes to find and purchase the right product significantly affects user satisfaction; hence, the ability to save time motivates customers to recurrently use voice intelligence throughout the value chain.

#### Advantages and challenges of voice intelligence

Despite the environments and domains to which voice intelligence is applied, the perceived convenience and enhanced user experience are deemed to be the most notable advantages of voice intelligence.

First, the perceived convenience during the human brain's decision-making process is an important reason for this wide incorporation (Shugan, 1980; Kemp, 2019). Convenience is described as experiencing minimal effort or barriers during activities (Nass and Brave, 2005). The human brain wants to make quick decisions, especially for low involvement purchase, without processing too much information (Klaus and Zaichkowsky, 2020). Self-learning algorithms enable voice intelligence to design a personalized user profile based on a user's purchase history and user activity by excluding interferences of low-involvement purchases. This possibility for shortening value chain processes through voice intelligence thus increases users' experienced convenience (Klaus and Zaichkowsky, 2020). Compared to the “old-fashioned” value chain that combines visual and touch elements, this convenient new “zero-touch” consumer purchasing behavior is revolutionary (Kinsella, 2019).

Delivering a high level of convenience is crucial for consumer loyalty and company valuation (Porcheron et al., 2018). This increase in loyalty and valuation is substantiated by consumer willingness to pay a significant percentage (up to 91%) more for experiencing the ease of receiving home delivery meals (Chen et al., 2020). Accordingly, performing multiple tasks simultaneously through voice interaction while saving physical and cognitive time leads to more convenience through flexibility and potential usability, which forms a practical tool for assisting the growing societal eagerness for multitasking (Moorthy and Vu, 2015).

Another benefit of voice intelligence is the enhanced user experience. Voice user interface has an essential role in perceived user experience through verbal communication (Oberoi, 2019). Verbalizing information is a distinctive anthropomorphic characteristic of voice intelligence that leads to an improved user experience (Porcheron et al., 2018). Anthropomorphism is the assigning of human attributes to non-organic things (Duffy, 2003). The verbalization of information is the reason why voice interaction is seen as more human, as voice effectuates a stronger social presence and more personification (Bartneck and Okada, 2001; Nass and Lee, 2001). Accordingly, oral interaction between users and technology leads to a more natural and productive conversation, as voice output results in a higher degree of perceived user credibility and competence (Chérif and Lemoine, 2019).

Meanwhile, voice intelligence faces several major challenges. These barriers have internal and external causes, which form decisive factors to its incorporation. Voice intelligence's first challenge is social acceptability (Efthymiou and Halvey, 2016; de Barcelos Silva et al., 2020). Social acceptability affects users' willingness to interact with technology. This willingness depends on three factors: the search case, the audience, and the user's location (Efthymiou and Halvey, 2016). Interacting with technology to execute a search case depends heavily on the environment and audience (Rico and Brewster, 2010; Moorthy and Vu, 2015). Accordingly, being with relatives positively affects the user's perceived social acceptability for interacting with voice intelligence, especially when sharing personal information (Rico and Brewster, 2010; Efthymiou and Halvey, 2016).

The second challenge is the perceived privacy issue while interacting (Alepis and Patsakis, 2017; McLean and Osei-Frimpong, 2019). To deliver a seamless user experience, voice software needs to have a significant amount of linguistic data input to learn and to adjust its interaction capability (Zhu et al., 2014). Accordingly, the intended constant interaction helps voice intelligence to improve its algorithms and excel in its role (Ezrachi and Stucke, 2017). This feature also means that voice-based assistants obtain more personal information than any other technology. Therefore, the continuous interaction possibility forms a privacy challenge that requires the rethinking of current privacy laws (McLean and Osei-Frimpong, 2019).

The third challenge is the perceived security risk. Voice interaction is a basis of biometric technology (Mahfouz et al., 2017). Biometric technology comprises an automated identification method which uses physiological or behavioral characteristics (Quatieri, 2002). Voice recognition verifies users by recognizing sound patterns as a unique authorizer for multiple systems (Rashid et al., 2008). This type of verification is safer than older password methods, as the physiological characteristics of voice patterns are difficult to alter (Hanzo et al., 2000). Nevertheless, using voice as a means of authorization also allows malicious actors to obtain private information through harmful applications (Alepis and Patsakis, 2017). The constantly active modus of voice intelligence gives multiple applications, including harmful variants, access to communication channels and sensitive information without the user's knowledge. Accordingly, the current biometric model results in security problems that challenge an individual's perception of voice intelligence technology.

### Social-technological factors in HCI

HCI is crucial to incentivizing individuals to use technology to complement, unburden and improve their daily lives (de Boer and Drukker, 2011; Jokinen, 2015). This subsection reviews the underlying factors, namely human emotions and user perceptions, associated with HCI and their influences on the level of adoption of voice intelligence.

#### Human emotions, user perceptions, and technology adoption

In recent decades, human emotions have captured increased attention in relation to technology adoption (Hassenzahl and Tractinsky, 2011). Human emotions comprise a reaction triad on external and internal components, consisting of subjective feelings, neurophysiological response patterns and motorial expressions (Johnstone and Scherer, 2000). This emotional triad is crucial to understanding human incentives for using technology (de Boer and Drukker, 2011).

Past studies on HCI have focused on its efficiency (Thüring and Mahlke, 2007; Mahlke and Minge, 2008). However, there is a growing idea that emotional reactions and enjoyability during interactions impact the user experience (Hassenzahl and Tractinsky, 2011). The appraisal theory introduced by Scherer (2009) further substantiated this trend of research (Jokinen, 2015). The theory entails primary and secondary appraisals (Lazarus et al., 1970; Lazarus, 2001). The primary appraisal evaluates a situation through personal goals and values (Lazarus, 2001). The secondary appraisal comprises the user's control and ability to adjust to specific events (Jokinen, 2015). Both appraisals indicate user willingness and motivation to interact with technology; hence, it is beneficial to incorporate the appraisal theory as a base for this research, considering the voice intelligence adoption process.

Confidence is one of the essential emotional states that influences a user's attitude toward and frequent usage of technology (Gardner et al., 1993). The Mobile Phone Technology Adoption Model (MOPTAM) identified that perceived ease of use and perceived usefulness are two vital influential factors of user confidence (van Biljon and Kotzé, 2004; Wong and Hsu, 2008). Perceived ease of use is defined as the experience of minimal physical or mental effort when using a technology (Davis et al., 1989). According to Hackbarth et al. (2003), perceived ease of use can be improved by increasing an individual's system experience. Therefore, decreasing user friction while interacting with voice intelligence leads to increased confidence and a higher perceived ease of use (Davis et al., 1989; Wang, 2015).

Furthermore, perceived usefulness is defined as an individual's perception that using technology increases performance (Davis et al., 1989). This increase in performance is effectuated by choosing the most suitable technology to efficiently execute and finalize the intended tasks (Chitturi et al., 2008; Kim et al., 2016).

Another emotional state that influences HCI is user trust (Zhang et al., 2019). Trust comprises a person's ability in and benevolence toward using technology, as well as how the user interprets a system's functionality (Szumski, 2020). The perceived security risks have a substantial influence on user trust in technology (Alford, 2020; Szumski, 2020). The main reasons for distrust in voice security measures are the single authentication systems (Wu et al., 2015). Still, voice intelligence interaction is primarily effectuated by using a unimodal biometric system (Dimov, 2015; Thakkar, 2021). The authentication process within unimodal systems uses a single biometric characteristic as digital information key for user validation and verification (Oloyede and Hancke, 2016). Unfortunately, this way of interacting is vulnerable to false interpretations (Zhang et al., 2019). Security risks in voice intelligence comprise the misinterpretation and impersonation of voice input (Wu et al., 2015). Two types of malignant functions cause security risks that affect voice intelligence perception: voice squatting and voice masquerading (Zhang et al., 2019). Voice squatting exploits different ways of placing a request and variations in pronouncing the action phrase (Brewster, 2017). External threats can easily incorporate the names of multinationals in action phrases, which are then linked to accompanied applications (Zhang et al., 2019). Furthermore, voice masquerading focuses on the sequence structure in voice commands (Brewster, 2017). A pernicious form of malware uses masquerading to gather sensitive information by quietly continuing to operate after pretending to hand over the control to the next application. Therefore, perceived security risks have been identified as the third influential factor of the adoption of voice intelligence in this research.

Privacy concerns, the second substantial factor, affect trust perception (Moorthy and Vu, 2015; Condliffe, 2019). Voice intelligence is used widely and therefore requires many software permissions (Alepis and Patsakis, 2017). An individual's privacy can be harmed because of information gathered without his or her knowledge (Collier, 1995). Because of the intensified interaction, cloud-native voice intelligence data contains more personal and sensitive information than predecessor technology (Cho et al., 2010). Accordingly, malintended individuals can use this data for harmful actions (Cho et al., 2010; Wu et al., 2015). This fear for privacy vulnerability has a negative influence on perceived trust of voice intelligence (Moorthy and Vu, 2015). Accordingly, perceived privacy concerns have been identified as the fourth influential factor for the adoption of voice intelligence in this study.

#### Anthropomorphism and user perceptions

Perceived social benefit, which relies on creating a social entity (McLean and Osei-Frimpong, 2019), is deemed to be a key factor of user perception (Chitturi et al., 2008). Creating a social entity is effectuated by merging technological and social characteristics (Moussawi et al., 2020). Speech is crucial during interactions, as it gives essential insights into personality and intentions (Edwards et al., 2019). Accordingly, HCI through voice can be adjusted significantly by applying social rules like politeness and courtesy to the AI device during a dialogue (Moon, 2000). This familiar mannerism during interaction drives users to allocate human-like characteristics to the device, such as expertise and gender (Edwards et al., 2019). This anthropomorphic tendency evokes social presence and attractiveness, leading individuals to experience a higher willingness to interact with AI technology in the same way as they do with others. As a result, users become comfortable during conservations, forming an emotional connection with the AI entity (Cerekovic et al., 2017).

Anthropomorphism is a user's willingness to allocate human emotional characteristics to non-organic agents (Verhagen et al., 2014). It has become imperative in the research on HCI interface design, as it is a promising influential factor of AI adoption (Li and Suh, 2021). Three main anthropomorphic research streams have been identified. The first emphasizes the positive effect of anthropomorphism on technological trust and perceived enjoyability in autonomous vehicles and on intelligent speaker adoption (Waytz et al., 2014; Wagner et al., 2019; Moussawi et al., 2020). The second stream reveals the positive influence of anthropomorphism on user adoption of chatbots and smart-speakers in the consumer journey by enhancing user enjoyment and trust (Rzepka and Berger, 2018; Moussawi et al., 2020; Melián-González et al., 2021). The third stream highlights the positive effect of emotional factors on trust and service evaluation and how language variation affects this user perception (Choi et al., 2001; Qiu et al., 2020; Toader et al., 2020), discussing the influences of anthropomorphism on various aspects of AI technology. All these research streams confirm the significant effect of anthropomorphism on user perception of technology.

Nevertheless, finding the right technological improvements that do not interfere with user experience is challenging, due to the novelty of zero-touch interfaces. Prior research on website personality, mobile interface personality and brand personality illustrated the most prominent similarities of technical capabilities and user interaction to voice intelligence based on user experiences (Aaker, 1997; Chen and Rodgers, 2006; Johnson et al., 2020). Four characteristics have been identified as having potentially significant effects: functional intelligence, sincerity, information creativity, and applicable voice tone and intonation (Kinsella, 2019; Poushneh, 2021).

The first of these, functional intelligence, is the level of effectiveness, usefulness and reliability generated to answer or perform a given request (Pitardi and Marriott, 2021). This capability increases the technology's reliability and improves user perception (Waytz et al., 2014), resulting in an individual gaining trust and confidence in using technology for task completion processes.

Second, sincerity is defined as honesty and genuineness toward social entities (Johnson et al., 2020). Like functional intelligence, sincerity allows an individual to experience a higher level of control. Perceived control during the interaction is stimulated by the device's adoption of a submissive attitude toward the user (Exline and Geyer, 2004). This characteristic results in a higher sense of user control during the interaction, which creates an incentive to intensify the human-technology relationship (Stets and Burke, 1996). Therefore, positioning the user as the dominant entity during the interaction results in the user experiencing more trust and enjoyment when operating the device.

Third, information creativity of voice intelligence can be defined as combining both novel and informative elements in a helpful response (Zeng et al., 2011). Piffer (2012) clarified creativity by introducing a three-dimensional framework that measures the novelty, usefulness and impact of products or information. As a result, information used in responses formulated by voice intelligence needs to comply with every dimension before it can be assessed as “creative”. This compliance is essential, as the perceived degree of creativity affects users' interest in learning more about technological capabilities and motivates regular use (Poushneh, 2021).

Fourth, the tone of voice determines the feelings a group of words gives when a message is communicated (Sethi and Adhikari, 2010). Choosing a tone that fits the situation is crucial for perceiving user satisfaction. For example, a humoristic voice tone does not work with a profound elaboration of a financial rapport (Moran, 2016). Users experience higher satisfaction when computers have a specific gender (Schwär and Moynihan, 2020). Therefore, changing the tone of voice depending on the activity can positively affect the adoption of voice intelligence in the customer value chain. In addition, according to the given request, adjusting the correct voice intonation can significantly affect an individual's experienced confidence and trust in both the evaluation and purchase phases in the consumer journey (Moran, 2016).

According to the discussion above, when these anthropomorphic characteristics are properly embedded in the interface design of the voice intelligence, user perception toward the voice intelligence is enhanced. First, the application of anthropomorphic characteristics reduces a tasks' difficulty by increasing trust in the technology (Gardner et al., 1993; Kinsella, 2019), resulting in a higher level of perceived ease of use. Thus, the first hypothesis is constructed as follows:

***H***_**1**_***:*** *Anthropomorphic characteristics are likely to increase users' perceived ease of use, which in turn positively influences the adoption of voice intelligence in the customer value chain*.

Second, incorporating functional intelligence and information creativity as additional voice intelligence output leads to a more informative and valuable interaction between user and machine (Pitardi and Marriott, 2021). These anthropomorphic characteristics give a user more context in addition to the actual responses. When voice intelligence transforms the output into messages with more valuable information, users can benefit from higher user productivity and improved user performance (van Biljon and Kotzé, 2004). Accordingly, the second hypothesis of this study is constructed as follows:

***H***_**2**_***:*** *Anthropomorphic characteristics are likely to increase users' perceived usefulness, which in turn positively influences the adoption of voice intelligence in the customer value chain*.

Third, creating a social entity by implementing anthropomorphic characteristics, such as a formal voice intonation, improves user perception toward the technical competence of a voice intelligence device. In turn, applying the proper anthropomorphic characteristics transforms the human-machine interaction into a more trustworthy process (Moller et al., 2006). This increase in trust eventually reduces users' perceived security risk when they use voice intelligence for relational exchanges. Thus, the third hypothesis is established as follows:

***H***_**3**_***:*** *Anthropomorphic characteristics are likely to decrease users' perceived security risks, which in turn positively influences the adoption of voice intelligence in the customer value chain*.

Fourth, humanizing the interaction with technology helps to strengthen the user relationship and results in more perceived control and reliability (Exline and Geyer, 2004; Pitardi and Marriott, 2021). The higher level of perceived control and reliability mitigates users' privacy concerns and thus increases their trust toward the interaction with the computer, which has a decisive influence on their recurring usage of the technology (Waytz et al., 2014). Therefore, the fourth hypothesis is the following:

***H***_**4**_***:*** *Anthropomorphic characteristics are likely to decrease users' perceived privacy concerns, which in turn positively influences the adoption of voice intelligence in the customer value chain*.

#### Multimodal biometric authentication and user perceptions

User identification plays a vital role in the adoption of technologies (Bhattacharyya et al., 2009; Zhang et al., 2019). The technological world is traditionally protected by security codes and “traditional” hardware keys. These security measures are vulnerable to malicious activities (Mahfouz et al., 2017). Biometric authentication solves this problem by using human characteristics as verification (Kinsella, 2019). This identification method is safer because of the uniqueness of an individual's characteristics, which are significantly less vulnerable to harmful activities (Zhang et al., 2019).

The biometric authentication process starts with the enrollment phase, in which the user shares his or her biometric data for the first time (Jain et al., 2004). These biometrics are analyzed by the system and separated as distinctive features. While filtering the data, the system builds a features template based on the identification characteristics (Liu et al., 2017). The recognition phase starts after the enrollment is finalized. This second phase compares re-acquired biometric data with the stored feature template (Jain et al., 2004). Table 2 shows the technical modules used for the recognition and identification of the characteristics in both phases (Mahfouz et al., 2017). The system ends the process by generating a similarity score. A higher matching score indicates a better similarity between the two datasets (Liu et al., 2017).

**Table 2:** Technical modules of the biometric authentication process (Liu et al., 2017; Mahfouz et al., 2017).

**Modules**	**Names**	**Functions**
1	Sensor	Measures or records the unprocessed biometric data of the user.
2	Biometric extractor	Cleans the raw data by detecting and removing oddities to improve the data quality.
3	Biometric matcher	Compares the input features with the data template to generate a matching score.

Deloitte reported that biometric authentication has attained critical mass as a safe and convenient identification method (Westcott et al., 2018). Biometric authentication can serve as a faster, more convenient, and practical user identification method, by which the system does not need to ask for any non-natural interference. Subsequently, this shorter and quicker authentication process improves the experienced utilitarian benefits (Wang, 2015; Rauschnabel et al., 2018), particularly ease of use and usefulness, and the overall human-technology relationship (McLean and Osei-Frimpong, 2019).

A reason for the increase in biometric authorization is the growing consumer awareness of data breaches and personal data theft (Westcott et al., 2018). Developments in biometrics answer this growing demand by delivering a more personal and robust identification option. Biometric systems have gained higher acceptance levels by ensuring user security and privacy (Jain et al., 2004). Accordingly, the use of biometric authentication mitigates users' perceived security risks and privacy concerns regarding technological capabilities to safely execute relational exchanges (Mahfouz et al., 2017).

In turn, these prior studies demonstrated the desired effect of biometric characteristics on the perceived ease of use, usefulness, security risks and privacy concerns, stimulating the adoption of technologies.

However, the overall number of available (unimodal) authentication systems still rely on only one biometric information source: either voice or facial. This reliability on one characteristic could lead to authorization problems caused by noisy data (Thakkar, 2021). In voice intelligence, voice recognition is the most dominant biometric authentication to date. However, using voice recognition for relational exchanges is susceptible to malicious activities (Dimov, 2015; Wu et al., 2015). Downloading anti-malicious software packages does not provide full security measures. The use of facial recognition as a second physiological authorization method in addition to voice recognition could improve the security of user privacy and personal data and thereby raise user trust (PWC, 2018). Thus, combining two characteristics, namely voice and facial recognition, in an authentication method solves the limitations of a unimodal biometric system. This combination consolidates data from different sources to compensate for the limits of either characteristic (Jain et al., 2004). Accordingly, a multimodal authentication system that combines two different biometric features is suggested.

Voice input and facial recognition are biometric characteristics with a significant and advanced role in “zero-touch” HCI (Liu et al., 2017). Combining both physiological attributes in the authentication process results in a 50% reduction of the error rate compared to using one or the other unimodal biometric system independently (Hazen et al., 2003). Consequently, expanding a unimodal system into a multimodal variant significantly improves the user journey within the human-machine interaction.

To be specific, multimodal biometric authentication enhances user perception and positively influences the adoption of voice intelligence in the customer value chain. First, incorporating a multimodal biometric authentication system into voice intelligence shortens and accelerates the authentication process, which improves the perceived ease of use (Mahfouz et al., 2017). In turn, compared to the previous identification methods, this shorter and quicker authentication process strengthens overall rapport between the user and voice assistant, thus increasing the likelihood that a user will adopt voice intelligence. Therefore, the fifth hypothesis is constructed as follows:

***H***_**5**_*: Multimodal biometric authentication is likely to increase users' perceived ease of use, which in turn positively influences the adoption of voice intelligence in the customer value chain*.

Second, using multimodal biometric characteristics leads to not only a more convenient but also a safer authentication process than its predecessors (Kinsella, 2019). This improvement is effectuated by offering a higher level of continuity and transparency in the customer value chain (Mahfouz et al., 2017). Accordingly, users perceive a higher level of usefulness, which comprises the idea that machine interaction increases actual productivity and thus leads to a higher level of adoption (Davis et al., 1989). Accordingly, the sixth hypothesis is proposed as follows:

***H***_**6**_***:*** *Multimodal biometric authentication is likely to increase users' perceived usefulness, which in turn positively influences the adoption of voice intelligence in the customer value chain*.

Third, a unimodal authentication system that focuses on only one biometric characteristic is vulnerable to security risks due to noisy data (Thakkar, 2021). Implementing a multimodal biometric authentication system with both voice and facial recognition mitigates this perceived security risk by reducing the error rate of noisy data (Hazen et al., 2007). This reduction in perceived security risk results in a safer and improved user experience of a relational exchange, in turn increasing users' willingness to adopt the technology. Accordingly, the following hypothesis is formulated:

***H***_**7**_***:*** *Multimodal biometric authentication is likely to decrease users' perceived security risk, which in turn positively influences the adoption of voice intelligence in the customer value chain*.

Fourth, additional measures embedded in the multimodal biometric authentication offer a personalized authorization process in the customer value chain, strengthening users' trust in the technology's ability to effectively handle privacy concerns. This strengthened trust in technology based on reduced privacy concerns in the customer value chain will eventually result in a more diverse and frequent use of voice intelligence (Condliffe, 2019). Therefore, the final hypothesis is constructed as follows:

***H***_**8**_***:*** *Multimodal biometric authentication is likely to decrease users' perceived privacy concerns, which in turn positively influences the adoption of voice intelligence in the customer value chain*.

## Research method

We carried out a 2 × 2 online experiment in April 2021 for four main reasons. First, an experiment allowed us to better examine the association of multiple influential factors and the adoption of voice intelligence by having the possibility to control cofounding variables and to measure and eliminate the tertium quid (Field and Hole, 2003).

Second, in this research, we have two conditions, namely anthropomorphism and multimodal biometric authentication, that simultaneously manipulate the subjects. Therefore, experimentation is the desired research method (Field and Hole, 2003; Haerling Adamson and Prion, 2020).

Third, a 2 × 2 factorial design allowed us to efficiently compare parallel manipulations (Hearling and Prion, 2020), making it possible to cross both predictors to determine the main and interaction effects (Landheer and van den Wittenboer, 2015; Asfar et al., 2020).

Fourth, using Internet as the medium, we were able to reach larger and diverse samples with limited financial costs. The web-based design was especially helpful when a physical laboratory experiment was not possible during the pandemic.

A pilot study, with 12 subjects per condition (Julious, 2005), was conducted before the actual experiment to test feasibility, and to determine and forecome potential consequences (Thabane et al., 2010).

### Treatment design

We manipulated two experimental conditions, namely *with* and *without*, for anthropomorphic characteristics and multimodal biometric authentication, respectively. Table 3 exhibits the four conditions, including (T1) without anthropomorphic characteristics and multimodal biometric authentication; (T2) without anthropomorphic characteristics but with multimodal biometric authentication; (T3) with anthropomorphic characteristics but without multimodal biometric authentication; and (T4) with anthropomorphic characteristics and multimodal biometric authentication.

**Table 3:** 2 × 2 Factorial design.

**Independent variables**		**Multimodal biometric authentication (MBA)**	
		**With**	**Without**
Anthropomorphism (ANT)	With	T4: ANT | MBA	T3: ANT | NMBA
	Without	T2: NANT | MBA	T1: NANT | NMBA

The four conditions were materialized in four short audio files. Each lasted approximately 3 min and consisted of a conversation between a voice assistant called “Iris” and its user (see Appendix I in Supplementary material). During this dialogue, the user executed multiple tasks in both customer value chain phases. Each phase included a voice intelligence process in which “Iris” used cloud-based text-to-speech and speech-to-text services as initial response for each activity. Accordingly, the voice assistant called “Iris” was used as a tool to let the user interact with voice intelligence during different tasks.

During the evaluation phase, the user executed tasks which resembled straight-forward recurring activities, such as checking the weather forecast and adding items to a shopping list. As argued by Kemp (2019), for recurring system usage, it is essential to improve the friction-time trade-off through process optimalization by shortening the execution time of simple tasks, such as checking the weather results, in a higher perceived convenience.

After the evaluation activities, the user performed multiple transactions within the purchase phase of the customer value chain. During these activities, the user ordered the voice intelligence software to purchase the items from the previously created shopping list in the evaluation phase.

Subsequently, the user decided to transfer money between banking accounts and perform a banking transaction to another person with voice intelligence.

Accordingly, each treatment consisted of different manipulations for each customer value chain phase applicable to their design. The audio files were created with help from the Amazon developers' community.

To manipulate the treatment of anthropomorphic characteristics, we took into consideration the four main characteristics that were identified as having potentially significant effects on user perception (see Section Anthropomorphism and user perceptions).

First, functional intelligence within “Iris” was expressed by formulating a helpful solution with an extra touch. This extra touch of functional intelligence delivers a valuable and efficient output applicable to the given command (Pitardi and Marriott, 2021). Functional intelligence was expressed during voice interaction by delivering an answer to a request followed by additional information applicable to that particular situation (Chen and Rodgers, 2006).

Second, sincerity within audio input comprises the technological capability to deliver a response that expresses honesty, friendliness, and humbleness toward users (Aaker, 1997). This anthropomorphic characteristic was manipulated through audio by expressing the supportive and modest role of “Iris” during the human-technology interaction (Aaker, 1997; Exline and Geyer, 2004). The modest attitude during the manipulation was expressed by asking the user if (s)he would like to receive more information about a requested topic (Exline and Geyer, 2004).

Third, the presence of information creativity within audio comprises the ability to deliver entertaining and bright information as attractive output (Poushneh, 2021). Information creativity was incorporated in voice intelligence through delivering an enthusiastic and helpful response applicable to the given situation (Zeng et al., 2011; Poushneh, 2021).

Last, fluctuating voice intonation was manipulated by incorporating an enthusiastic or causal response within the evaluation phase (Moran, 2016) and a formal response during the purchase phase in the customer value chain (Brandt, 2017). Accordingly, “Iris” will adapt an enthusiastic or causal response during simple daily tasks and formulates formal output when the user starts to perform financial transaction or purchase activities.

Voice recognition formed the dominant unimodal authenticator within voice intelligence. However, this dependance on a single information source suffered from authentication issues and problematic performances in real world applications (Wu et al., 2015). Multimodal biometric systems combine biometric information from two independent sources for validation purposes (Oloyede and Hancke, 2016). Accordingly, a second information source was added to the existing verbal validation process of voice intelligence. Voice interaction is predominantly executed through mobile phone use within a unimodal environment (PWC, 2018). Face identification in mobile technology formed the second biometric characteristic that perceived the zero-touch experience (Liu et al., 2017). Appending face recognition as second independent identifier transformed the existing unimodal system of voice intelligence into a new multimodal variant. Accordingly, the effect of multimodal biometric authentication was manipulated by incorporating an extra face recognition step (Pieraccini, 2012). The user was asked to first scan additional facial characteristics before the software accepted linguistic traits as validation during specific activities. This additional authentication was applied during the purchase phase in the customer value chain.

### Subjects and incentives

Amazon MTurk was used as a crowdsourcing platform to recruit subjects. By using MTurk, it was possible to filter the subjects against our criterion: subjects were required to have access to a mobile Internet connection (Singh, 2018). This is because voice intelligence is predominantly installed and used in portable devices, such as smartphones and tablets, making stationary and office-based settings inapplicable to this technology (PWC, 2018). To enhance the validity of the responses from the subjects, we further required the subjects to have the relevant user experience.

A subject who passed the given requirement was offered a small incentive of USD 0.40 to participate in one of the four conditions. A subject was allowed to participate in the experiment only once.

This research adopted a between-subjects design. Compared to a within-subject design, it minimized the learning and transfer effect across conditions, shortened the length of a treatment session and made subject randomization manageable (Suresh, 2011; Allen, 2017; Budiu, 2018).

### Experiment procedure and measurement of variables

Subjects were randomly assigned to one of the four conditions. When the experiment started, the subject received a vignette. This vignette consisted of two parts. The first part presented the purpose of this study and asked the subject's permission to proceed further. The second part introduced and explained voice intelligence technology to help the subjects form an equal basic understanding of the technology (Gourlay et al., 2014). To ensure that all subjects had read the vignette, participants were not able to click on the next-step button before a specific time period had elapsed.

Subsequently, the subject was asked to listen to a conversation between “Iris” and its user, representing one of the experimental conditions. Afterwards, the subject was required to fill in an online questionnaire, which measured the subject's perception toward the technology and his or her willingness to adopt it. Finally, at the end of the questionnaire, the subject was asked to answer demographic questions, which helped us delineate subgroups within the samples (Field and Hole, 2003).

The measurement items for each construct in the research model was adopted or adapted from the existing literature (see Appendix II in Supplementary material). The items were measured on a seven-point Likert scale (Strongly Disagree—Strongly Agree). A neutral alternative was also added to the scale to increase measurement quality (DeCastellarnau, 2018).

## Analysis and results

We obtained a total of 266 observations. Two attention checks were incorporated in the questionnaire to ensure participant attention. The first attention check required the subject to answer the control question: “Select option 5: Somewhat agree”. The second attention check asked the subjects to turn in a unique code after finishing the questionnaire. This unique code was given after the demographic question. Responses that failed either attention check were excluded from the dataset. Accordingly, we identified 240 valid observations, or 60 per treatment, for further analysis.

### Overview of subjects

Table 4 shows the demographic profiles of subjects in this study. Male subjects (69.2%) and subjects aged between 18 and 34 (57.9%) were the major ones in the sample. The majority of all respondents were highly educated; almost 90% of subjects held Bachelor or Master degrees. Since we sourced subjects *via* Amazon MTurk, an American crowdsourcing platform, over 80% of subjects in this study were residents of North America.

**Table 4:** Overview of the subjects.

**Characteristics**	**Frequency**	**Percentage**
Gender		
Male	166	69.2
Female	73	30.4
Non-binary	1	0.4
Age–groups		
18–24	12	5
25–34	139	57.9
35–44	60	25
45–54	20	8.3
55–64	9	3.8
Educational background		
High-School graduate	13	5.4
Attended college	14	5.8
Bachelor's degree	145	60.4
Master's degree	68	28.3
Continent		
Africa	2	0.8
Asia	29	12.1
Europe	5	2.1
North America	198	82.5
South America	6	2.5
Total	240	100

### Reliability and validity

Reliability and validity tests were performed to evaluate the measurement model. Cronbach's alpha and composite reliability tests were performed to measure the internal consistency of the scale items. Table 5 shows that Cronbach's alpha values and the composite reliability scores of all the variables were higher than 0.6 and 0.7, respectively. These results substantiate the internal consistency of the scale items in this study (Hair et al., 2011, 2019; Hamid et al., 2017).

**Table 5:** Assessment of measurement model.

	**Nr. of Items**	**Cronbach's alpha**	**Composite reliability**	**AVE**	***EU***	***US***	***PC***	***SR***	***AVI***
PEU	5	0.74	0.93	0.67	***0.82***				
PU	5	0.72	0.90	0.63	*0.71*	***0.80***			
PPC	5	0.73	0.91	0.67	*0.35*	*0.31*	***0.82***		
PSR	5	0.62	0.83	0.53	*0.46*	*0.53*	*0.56*	***0.73***	
AVI	5	0.74	0.92	0.63	*0.69*	*0.68*	*0.36*	*0.59*	***0.82***

PEU, perceived ease of use; PU, perceived usefulness; PPC, perceived privacy concerns; PSR, perceived security risks; AVI, adoption of voice intelligence. Diagonal elements (bold) are the square root of the AVE for each construct; Off-diagonal factors correspond to construct intercorrelations. The italics values are used to determine the discriminant validity. All values exceed the corresponding intercorrelation.

To determine the construct validity, both convergent and discriminant validity tests (Peter, 1981) were carried out. Convergent validity was tested based on Average Variance Extracted (AVE) per variable. As shown in Table 5, all AVE scores exceeded the 0.5 rule of thumb (Fornell and Larcker, 1981), indicating a sufficient convergent validity of the variables (Hair et al., 2011).

Discriminant validity assessment was based on the difference between the square root value of AVE and the correlation of variables; the correlation between variables must be lower than the square root value of AVE (Hair et al., 2011). As shown in Table 5, the square root value of AVEs exceeded their corresponding intercorrelation.

### Descriptive statistics

Table 6 lists the descriptive statics of all the variables of user perception based on the total observations. These variables are: Perceived Ease of Use (PEU), Perceived Usefulness (PU), Perceived Security Risk (PSR) and Perceived Privacy Concerns (PPC). All variables in this study had an average score of 5. The PEU (M = 5.66, SD = 0.94) and PU (M = 5.53, SD = 0.93) had the highest overall mean. This means that subjects perceived the practical potential of voice interaction technology in their daily lives. Meanwhile, subjects expressed their concerns about the intensive use of voice intelligence by also giving high scores to PPC (M = 5.66, SD = 0.94) and PSR (M = 5.53, SD = 0.93). In general, subjects showed a moderate willingness to adopt voice intelligence (M = 5.48, SD = 0.97) in various daily activities.

**Table 6:** Descriptive statistics.

	**Mean**	**SD**	**Min**	**Max**
PEU	5.66	0.94	2.20	7.00
PU	5.53	0.93	2.20	7.00
PSR	5.33	1.01	1.60	7.00
PPC	5.19	1.17	1.00	7.00
AVI	5.48	0.97	2.00	7.00

Table 7 displays the descriptive statistics per treatment. The group treated with a combined anthropomorphic and biometric element had the highest level of PEU (M = 5.92, SD = 0.73) and PU (M = 5.79, SD = 0.80). However, their PPC (M = 5.36, SD = 1.4) was also relatively higher than any other group. The group with only anthropomorphic treatment had the lowest PPC (M = 5.10, SD = 1.01) and PSR (M = 5.12, SD = 0.78). This implies that the incorporation of anthropomorphic characteristics into voice intelligence may reduce users' concerns about perceived privacy concerns and security risks.

**Table 7:** Descriptive statistics per treatment.

**Treatments**	**PEU**	**PU**	**PSR**	**PPC**	**AVI**
T1 (*N* = 60)	5.33 (1.16)	5.30 (1.15)	5.18 (1.22)	5.11 (1.18)	5.23 (1.18)
T2 (*N* = 60)	5.80 (0.89)	5.65 (0.76)	5.52 (0.79)	5.22 (1.04)	5.62 (0.97)
T3 (*N* = 60)	5.59 (0.84)	5.39 (0.88)	5.12 (0.78)	5.10 (1.01)	5.40 (0.88)
T4 (*N* = 60)	5.92 (0.73)	5.79 (0.80)	5.50 (1.11)	5.36 (1.40)	5.66 (0.76)

T1, without anthropomorphic and multimodal biometric treatment; T2, without anthropomorphic but with multimodal biometric treatment; T3, with anthropomorphic but without multimodal biometric treatment; T4, with anthropomorphic and multimodal biometric treatment.

Overall, subjects perceived a higher level of ease of use and usefulness and were more willing to adopt the voice intelligence when anthropomorphic or multimodal biometric treatments were applied.

### Direct effects of anthropomorphism and multimodal biometric authentication on adoption

We first performed a factorial ANOVA to examine the direct effect of anthropomorphic and multimodal biometric characteristics and their interaction effects on user adoption of voice intelligence. Table 8 exhibits the analysis results. Both factors showed significant effects on users' willingness to adopt voice intelligence, while the interaction between them did not show a significant effect [*F*_(1)_ = 3.043, *p* > 0.08].

**Table 8:** Factorial ANOVA analysis of direct effects.

**Treatment**	**df**	**Mean square**	***F*-value**	**Eta squared**	**95% C.I**.
MBA	1	13.824	14.935^***^	0.06	[0.02; 0.11]
ANT	1	3.851	4.160^*^	0.02	[0.00; 0.05]
ANT | MBA	1	2.817	3.043	0.01	[0.00; 0.05]
Residuals	236	0.926			

* *p < 0.05*,

^**^p < 0.01,

*** *p < 0.001*.

The positive effect of multimodal biometrics [*F*_(1)_ = 14.935, *p* = 0.00] is higher than that of anthropomorphism [*F*_(1)_ = 4.160, *p* = 0.04]. Further examination of the effect size (see Table 8), according to the guidelines of Cohen (1988), revealed that the effect of multimodal biometrics was medium (η^2^ > 0.06), while the effect of anthropomorphism was marginal (η^2^ < 0.02).

In short, the factorial ANOVA indicated significant effects of both anthropomorphism and multimodal biometric authentication on users' willingness to adopt voice intelligence. The absence of an interaction effect suggests that these two predictors do not substantially affect each other.

### Hypotheses testing

Structural equation modeling (SEM) was performed to validate the hypotheses by using the LAVAAN-package in R-studio. The results of the path analyses are presented in Table 9. Anthropomorphism showed a significant impact on all the perceived factors, including PEU (β = 0.67, *p* < 0.05), PU (β = 0.40, *p* < 0.01), and PSR (β = −0.77, *p* < 0.05), except for PPC (β = −0.52, *p* > 0.24). It can be argued that the incorporation of anthropomorphic characteristics into voice intelligence increases users' perceived ease of use and perceived usefulness while reducing the perceived security risks toward voice intelligence in the customer value chain. Nevertheless, it did not significantly affect users' perceived privacy concerns.

**Table 9:** Path analyses.

**Paths**	**Est**	**SE**	**Z**	**95% CI lower**	**95% CI upper**
**Direct paths**					
ANT → PEU	0.6733333^*^	0.2865799	2.3495484	1.2331072	0.1045226
ANT → PU	0.4000000^**^	0.1545632	2.5879387	0.6966261	0.0896086
ANT → PSR	−0.7733333^*^	0.3559318	−2.1727007	−1.4400280	−0.0584524
ANT → PPC	−0.5199999	0.4442834	−1.170424	−1.3440642	0.4131769
MBA → PEU	−0.2533333	0.2968869	−0.8532991	−0.8383091	0.3273333
MBA → PU	−0.2933333	0.2860157	−1.0255845	−0.8418642	0.2946706
MBA → PSR	−0.0333333	0.3588537	−0.0928884	−0.6425856	0.7668297
MBA → PPC	−0.2800000	0.4486493	−0.6240955	−1.1246750	0.6332600
PEU → AVI	0.6490008^***^	0.0680327	9.5402193	0.5126917	0.7830255
PU → AVI	0.6645794^***^	0.0618023	10.8122354	0.5429231	0.7872081
PSR → AVI	−0.5641075^***^	0.0571241	−9.8810212	−0.4544264	−0.6781363
PPC → AVI	−0.3268894^***^	0.0626997	−5.2169322	−0.2184188	−0.465145
**Mediation**					
ANT → PEU → AVI	0.4198978^*^	0.1867468	2.2484875	0.8132560	0.0733127
ANT → PU → AVI	0.2459281^*^	0.0976730	2.5178720	0.4426606	0.0566385
ANT → PSR → AVI	0.4339686^*^	0.2187507	1.9838498	0.8881447	0.0338555
ANT → PPC → AVI	−0.1672892	0.1580518	−1.058445	−0.5145335	0.1149420
MBA → PEU → AVI	−0.1708457	0.2037402	−0.8385465	−0.5888469	0.2095889
MBA → PU → AVI	−0.2095392	0.2091822	−1.0017067	−0.6322932	0.1991155
MBA → PSR → AVI	0.0189017	0.2052428	0.0920943	−0.3873673	0.4192312
MBA → PPC → AVI	0.0929792	0.1570425	0.5920644	−0.4200953	0.1982651

* *p < 0.05*,

** *p < 0.01*,

*** *p < 0.001*.

On the contrary, the results did not reveal any significant influence of multimodal biometric authentication on users' PEU (β = −0.25, *p* > 0.39), PU (β = −0.29, *p* > 0.30), PSR (β = −0.03, *p* > 0.92) or PPC (β = −0.28, *p* > 0.53). This implies that the effect of multimodal biometric authentication on a user's adoption of voice intelligence is not through these four user perceptions.

The analysis further revealed that the direct effects of PEU (β = 0.65, *p* < 0.001), PU (β = 0.66, *p* < 0.001), PSR (β = −0.56, *p* < 0.001), and PPC (β = −0.33, *p* < 0.001) on user adoption of voice intelligence were all significant. This indicates that a higher level of perceived ease of use and perceived usefulness and a lower level of perceived security risks and perceived privacy concerns lead to an increased likelihood of users adopting voice intelligence in the customer value chain.

The mediation test confirmed the mediating role of PEU (β = 0.42, *p* < 0.05), PU (β = 0.25, *p* < 0.05) and PSR (β = 0.43, *p* < 0.05) between anthropomorphism and user adoption of voice intelligence. This signifies that incorporation of anthropomorphic characteristics into voice intelligence positively influences user adoption *via* perceived ease of use, perceived usefulness, and perceived security risks.

The SEM analysis used bootstrapping to create 10,000 resamples to assess the mediation effect. Bootstrapping is a superior method because it does not tend to systematically shift toward zero due to positively skewed values (Koopmans et al., 2014). Nevertheless, existing literature pointed out the importance of considering a possible high Type 1 error rate when applying bootstrapping (Fritz et al., 2012; Koopmans et al., 2014). A Type 1 error comprises the event of rejecting a true null hypothesis (Cohen et al., 2003). The sample size of this study leads to the possible increase in Type 1 error rate of bootstrapping; the probability of a fluctuating error rate above 5% starts to increase after the sample size exceeds *N* = 140 observations (Koopmans et al., 2014). Therefore, an additional Sobel test was utilized to substantiate the previous mediating testing results.

The additional Sobel tests results of the mediating effect. Similarly to the SEM analysis with bootstrapping, the Sobel tests demonstrated that PEU (*Z* = 2.28, *p* < 0.05), PU (*Z* = 2.52, *p* < 0.05), and PSR (*Z* = 2.12, *p* < 0.05) significantly mediate the effect of anthropomorphism on the adoption of voice intelligence.

According to the aforementioned analysis, anthropomorphism affects user adoption of voice intelligence through perceived ease of use, perceived usefulness, and perceived security risks, and hence hypotheses H_1_ –H_3_ were supported. Anthropomorphism does not influence users' perceived privacy, and multimodal biometric authentication does not affect any user perception. However, all of the types of user perception affect users' willingness to adopt voice intelligence. Therefore, hypotheses H_4_ –H_8_ were partially supported. Table 10 summarizes the results of the hypotheses testing.

**Table 10:** Validation of the hypotheses.

**Hypothesis**	**Status**
H_1_: Anthropomorphic characteristics are likely to increase users' perceived ease of use, which in turn positively influences the adoption of voice intelligence in the customer value chain.	Supported
H_2_: Anthropomorphic characteristics are likely to increase users' perceived usefulness, which in turn positively influences the adoption of voice intelligence in the customer value chain.	Supported
H_3_: Anthropomorphic characteristics are likely to decrease users' perceived security risks, which in turn positively influences the adoption of voice intelligence in the customer value chain.	Supported
H_4_: Anthropomorphic characteristics are likely to decrease users' perceived privacy concerns, which in turn positively influences the adoption of voice intelligence in the customer value chain.	Partially supported
H_5_: Multimodal biometric authentication is likely to increase users' perceived ease of use, which in turn positively influences the adoption of voice intelligence in the customer value chain.	Partially supported
H_6_: Multimodal biometric authentication is likely to increase users' perceived usefulness, which in turn positively influences the adoption of voice intelligence in the customer value chain.	Partially supported
H_7_: Multimodal biometric authentication is likely to decrease users' perceived security risks, which in turn positively influences the adoption of voice intelligence in the customer value chain.	Partially Supported
H_8_: Multimodal biometric authentication is likely to decrease users' perceived privacy concerns, which in turn positively influences the adoption of voice intelligence in the customer value chain.	Partially supported

### *Post-hoc* analysis

User perception in this study consists of four specific types, namely PEU, PU, PSR, and PPC. The average score of these four factors in each response was calculated to represent an overall user perception toward the voice intelligence. A factorial ANOVA analysis was subsequently performed to examine the effect of ANT and BA on user overall perception. Table 11 reveals that both ANT [*F*_(1)_ = 11.72, *p* < 0.001] and MBA [*F*_(1)_ = 28.27, *p* < 0.001], as well as their interactions [*F*_(1)_ = 6.50, *p* < 0.05], significantly influence user perception of voice intelligence.

**Table 11:** Factorial ANOVA analysis of effects of BA and ANT on users' overall perception.

**Treatment**	**Df**	**Mean square**	***F*-value**	**Eta squared**	**95% C.I**.
ANT	1	9.204	11.7173^***^	0.05	[0.01; 0.10]
MBA	1	22.204	28.2667^***^	0.11	[0.05; 0.17]
ANT | MBA	1	5.104	6.49788^*^	0.03	[0.00; 0.07]
Residuals	236	0.786			

* *p < 0.05*,

^**^p < 0.01,

*** *p < 0.001*.

Moreover, a multiple regression model was utilized to examine the impact of the demographic and control variables, in addition to the four mediators, on user adoption of voice intelligence. The results (see Table 12) show that the effects of all the mediators on user adoption remained significant. Additionally, having an age between 35 and 44 (β = 0.42, *p* < 0.05), Bachelor degree (β = 0.50, *p* < 0.01) or Master degree (β = 0.42, *p* < 0.05) were shown to be predictors of user adoption of voice intelligence. Gender and location of residence did not influence user behavior.

**Table 12:** Regression for demographic and control variables.

**Variables**	**Coefficient**	**Standard errors**
Users' perceptions		
PEU	0.56702^***^	0.06132
PU	0.46199^***^	0.06630
PSR	−0.30658^***^	0.05476
PPC	−0.33341^***^	0.03965
Gender		
Male	0.28640	0.59893
Female	0.10956	0.59927
Non-binary	-	
Age–groups		
18–24	0.23545	0.61486
25–34	0.17946	0.18464
35–44	0.41781^*^	0.19317
45–54	0.26196	0.22145
55–64	0.46221	0.28414
Educational background		
High-School graduate	0.48594	0.24902
Attended college	0.48660	0.46167
Bachelor's degree	0.50146^**^	0.18408
Master's degree	0.42443^*^	0.19303
Continent		
Africa	0.56992	0.42655
Asia	0.02466	0.11804
Europe	0.12591	0.29959
North America	0.1717	0.42655
South America	0.18115	0.26708
R sq adjust. (0.6372)	0.6491 (*0.677)*	
R sq adjust. change	0.0181	
F statistic	24.27^***^	
No. of observation	240	

* *p < 0.05*,

** *p < 0.01*,

*** *p < 0.001*.

The italic value shows the effect in the adjusted R squared after changing the included variables during the analysis.

These findings suggest that while location and gender do not affect user adoption of voice intelligence, users with a higher level of education are more likely to adopt voice intelligence in the customer value chain.

## Discussion

In this section, we further discuss the results drawn from the online experiment. Our study confirms the effect of perceived ease of use, usefulness, security risk, and privacy concerns on user adoption of voice intelligence. These findings are in line with prior research on the influence of different user perceptions on technology adoption (e.g., de Boer and Drukker, 2011; Jokinen, 2015; Moorthy and Vu, 2015; Zhang et al., 2019). However, our findings of the effect of anthropomorphism and multimodal biometric authentication show contradiction with our hypotheses and with existing studies. Therefore, our discussion focuses on these two influential factors.

### The effect of anthropomorphism

Our study confirms that when anthropomorphic characteristics are incorporated into voice intelligence, users are more likely to adopt the technology in the customer value chain, specifically the evaluation and purchase phases. This effect is largely indirect through users' perceived ease of use, perceived usefulness and perceived security risk This finding is in line with previous research. These existing studies contend that anthropomorphism enhances human-machine interaction (Waytz et al., 2014; Kinsella, 2019) by incorporating characteristics that represent a higher state of mind (Choi et al., 2001; Qiu et al., 2020; Toader et al., 2020), and thus further motivate users to more frequently and widely adopt the technology in their daily lives (de Boer and Drukker, 2011; Jokinen, 2015).

However, according to our research, the use of anthropomorphic characteristics does not influence user adoption of voice intelligence through users' perceived privacy concerns, because anthropomorphism does not affect perceived privacy concerns. This contradicts the literature on anthropomorphism that asserts a positive effect of humanizing the interaction with technology on mitigating users' privacy concerns by strengthening perceived control and reality (Exline and Geyer, 2004; Pitardi and Marriott, 2021).

A possible reason for this is the influence of demographic factors. Graeff and Harmon (2002) point out that consumers' privacy concerns vary among demographic market segments when they purchase online. They find that younger consumers are more aware of data collection and privacy risks. This may be due to their relatively high data literacy as “digital natives”. Other prior research also demonstrates a positive relationship between education and privacy concerns (Graeff and Harmon, 2002; Zhang et al., 2002). With more knowledge about the technology, data collection and risks, consumers may have more privacy concerns when they use the technology throughout the customer value chain.

In our experiment, almost 90 percent of the subjects were young, aged between 18 and 44, and highly educated, with either Bachelor or Master degrees. According to the aforementioned discussion, they may have had a better understanding of voice intelligence and its associated personal and sensitive information collection. This may have further engendered their higher awareness of the possible risks when interacting with voice intelligence in the customer value chain. As a result, in general, their perceived privacy concerns toward voice intelligence were salient (M = 5.19, SD = 1.17), despite the social presence and attractiveness evoked by the anthropomorphic characteristics.

### The effect of multimodal biometric authentication

Our research does not manifest the hypothesized effect of multimodal biometric authentication on users' perceived ease of use, usefulness, security risks or privacy concerns. This contradicts existing studies that demonstrate the significance and growing importance of biometric authentication in both user trust and user confidence during HCI (Gardner et al., 1993; Westcott et al., 2018). A possible reason is the influence of the friction–time trade-off (Guzman, 2018; Kemp, 2019). Kemp (2019) argues that the friction–time trade-off affects users' perceived ease of use and usefulness toward the technology. In their study on continuous multimodal biometric authentication (CMBA), Ryu et al. (2021) found that current biometric authentication systems predominantly focus on the user re-authentication process without giving enough attention to the user experience. These additional authentication steps require more time and effort from consumers during HCI in the customer value chain. When the authentication window time exceeds users' perceived benefits, consumers perceive no improvement, sometimes even experiencing a negative effect on ease of use and usefulness of the technology (El-Abed et al., 2010).

In our study, when multimodal biometric authentication was applied, in addition to voice recognition, subjects needed to take additional steps to complete the facial recognition for authentication in the purchase phase of the customer value chain. Compared to conditions with unimodal biometric authentication, this multimodal approach requires users to spend more time and make more effort. As a result, their perceived ease of use and usefulness toward the voice intelligence in the customer value chain are not likely to improve. The path analyses even indicate a negative, though not statistically significant, effect on perceived ease of use (β = −0.25) and usefulness (β = −0.29). This finding is similar to the study on CMBA (Ryu et al., 2021).

Moreover, although the results drawn from our study do not show significant statistical evidence to support the influence of multimodal biometric authentication on users' perceived security risks and privacy concerns, the path analysis exhibited the hypothesized association between them: implementing multimodal biometric authentication in voice intelligence reduces users' perceived security risks (β = −0.033) and privacy concerns (β = −0.280). The insignificant statistical analysis results may be due to the lack of an immersive experimental environment. In our experiment, subjects could only listen to a conversation instead of directly talking with “Iris”. This may have caused a different, inaccurate or even biased perspective toward the voice intelligence device. As a result, the measured perceived security risk and privacy concerns may not completely, accurately or precisely reflect the effect of multimodal biometric authentication.

Finally, according to the *post-hoc* analysis in Section *Post-hoc* analysis, both anthropomorphism [*F*_(1)_ = 11.72, *p* < 0.001] and multimodal biometric authentication [*F*_(1)_ = 28.27, *p* < 0.001] have an impact on overall user perception toward voice intelligence. The effect of multimodal biometric authentication (η^2^ = 0.11) is more significant than the effect of anthropomorphism (η^2^ = 0.05). Unlike the four specific user perceived factors, this finding shows results similar to those of previous research. Zhang et al. (2019) conclude that offering an authentication process that does not demand any touch interference by the user increases the overall experience during human-machine interaction. It can also be argued that while the use of multimodal biometric authentication may not significantly enhance any of the specific user perceptions, it has a striking influence on the aggregate level of user experience.

### The interaction effect of anthropomorphism and multimodal biometric authentication

**Figure 2:**
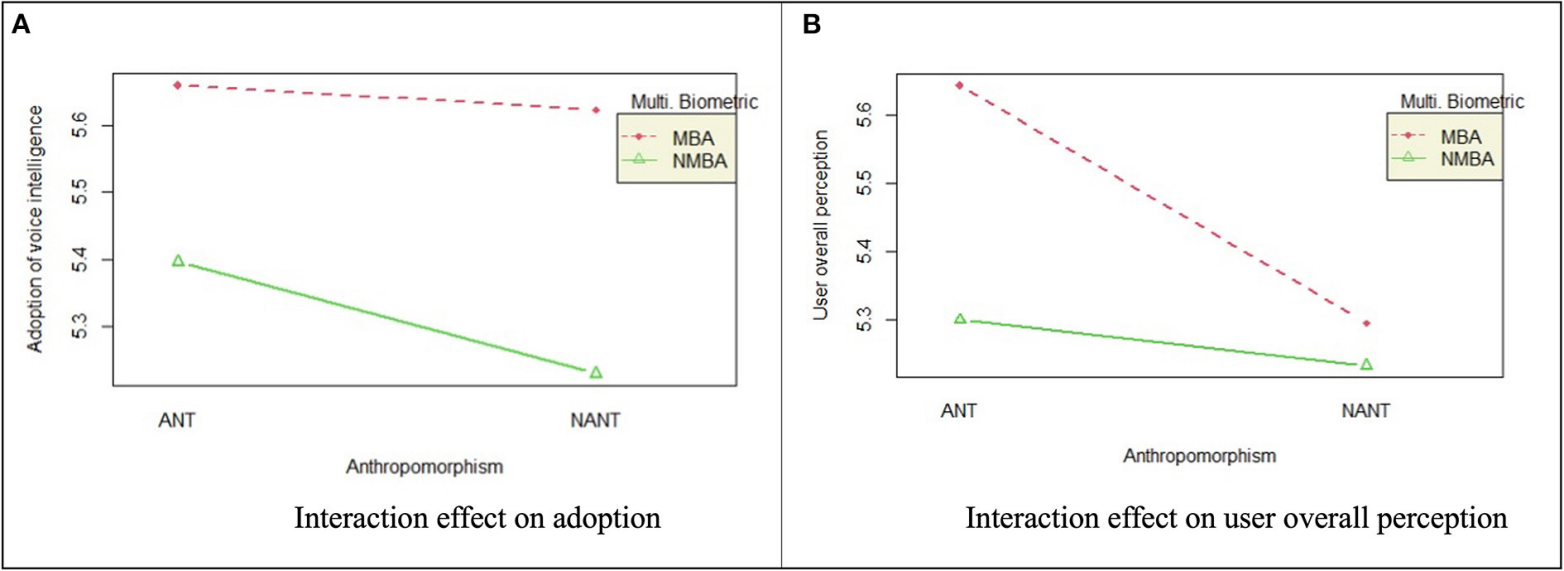
Interaction effects. **(A)** Examining the effect of anthropomorphism and multimodal biometric authentication on the customer's willingness to adopt voice technology in their value chain. **(B)** Examining the effect of anthropomorphism and multimodal biometric authentication on the overall customer perception of voice technology in general.

Our research does not reveal an interaction effect of anthropomorphism or multimodal biometric authentication on user adoption of voice intelligence. However, we have obtained some interesting findings based on their marginal interaction effect, illustrated by the evident non-parallel lines in Figure 2.

First, as can be seen from Figure 2A, the direct effect of anthropomorphism on user adoption of voice intelligence varies according to the presence or absence of multimodal biometric authentication. When multimodal biometric authentication is absent, the difference of user adoption between *with* and *without* anthropomorphic characteristics is more compelling than the situation when multimodal biometric authentication is present. This indicates that the effect of anthropomorphism on user adoption is influenced by multimodal biometric authentication.

Second, despite the presence or absence of anthropomorphic characteristics, the impact of multimodal biometric authentication on user adoption remains similar (see Figure 2A). This suggests that the influence of multimodal biometric authentication is barely affected by anthropomorphism.

Third, overall, users show most willingness to adopt voice intelligence when both features are applied, and least willingness when neither of these are present.

Regarding the effect of these two influential factors on overall user perception, an interaction effect is captured [*F*_(1)_ = 6.50, *p* < 0.05], though it is considered small (η^2^ = 0.03). Figure 2B illustrates that compared to the situation when multimodal biometric authentication is absent, the difference in overall user perception between *with* and *without* anthropomorphic characteristics is more remarkable when multimodal biometric authentication is present. This indicates that the effect of anthropomorphism on user overall perception is influenced by multimodal biometric authentication.

Similarly, when anthropomorphic characteristics are present, the difference in overall user perception between *with* and *without* multimodal biometric authentication is more noticeable than the situation when anthropomorphic characteristics are not incorporated.

Moreover, user perception reaches its highest level when both anthropomorphic characteristics and multimodal biometric authentication are applied to the voice intelligence device. On the contrary, when neither of these two features are incorporated, the level of user perception is lowest. This finding is consistent with prior research, which states that both anthropomorphism and multimodal biometric authentication affect user perception through visualizing and creating a more personalized and safer interaction (Jain et al., 2004; Waytz et al., 2014; Li and Suh, 2021).

Accordingly, it can be argued that the use of multimodal biometric authentication can leverage the advantages of incorporating anthropomorphic characteristics into voice intelligence. It further enhances users' perception toward the technology when they interact with voice intelligence in different phases of the customer value chain. As a result, users are more likely to adopt voice intelligence. Nevertheless, when anthropomorphic characteristics are absent in the interface design, the use of multimodal biometric authentication becomes of paramount importance in strengthening user perception and adoption of voice intelligence.

## Conclusion

In this section, we summarize the key findings of this study, present its contributions to the literature and business implications, and discusses its limitations and possible directions for future research.

### Summary of key findings

This paper examines the influence of socio-technological factors on the adoption of AI voice intelligence. We studied the impact of anthropomorphism and multimodal biometric authentication on user adoption of voice intelligence in the customer value chain. Our empirical findings reveal that the use of anthropomorphic characteristics and multimodal biometric authentication positively affects users' willingness to adopt voice intelligence through the enhanced overall experience of the interaction with the technology. Users' perceptions, especially perceived ease of use, usefulness, security and privacy, determine their willingness to adopt voice intelligence.

The effect of anthropomorphism on the adoption of voice intelligence is present specifically in perceived ease of use, usefulness and security risk. Multimodal biometric authentication directly affects users' adoption of voice intelligence. Although it does not have an influence on any specific perceived factors, at the aggregate level it improves overall user perception of the technology. Privacy concerns, nevertheless, are not significantly affected by either of the influential factors. This may be due to the demographic characteristics of the sample population; young and highly educated users generally have a high awareness of the privacy concerns of data collection and its possible risks.

When both anthropomorphism and multimodal biometric authentication are incorporated into the interface design, users' perception toward the human-machine interaction and their willingness to adopt voice intelligence reach the highest level.

### Theoretical contributions

This research makes several contributions to the literature on HCI and voice intelligence. First, we theoretically develop and empirically test the simultaneous impact of anthropomorphism and biometric authentication on user experience of interaction with machine and their adoption of the technology. Unlike prior research that was limited to either anthropomorphism (Choi et al., 2001; Waytz et al., 2014; Rzepka and Berger, 2018; Moussawi et al., 2020; Toader et al., 2020; Melián-González et al., 2021) or biometric authentication (Jain et al., 2004; Zhang et al., 2019), this paper examines their interaction effect, contributing to the further understanding of these two influential factors of HCI.

Second, this paper extends the existing literature on the impact of HCI on adoption of mobile technology. We identify the user perception factors of HCI based on the view of fundamental human emotions (de Boer and Drukker, 2011; Hassenzahl and Tractinsky, 2011; Jokinen, 2015) and their relevance to verbalized information-based technology (van Biljon and Kotzé, 2004; van Biljon and Renaud, 2008). Thus, this study also contributes to the literature on technology acceptance in the specific context of the use of natural languages.

With regard to voice intelligence, this study is one of the first to empirically test the effect of multimodal biometric authentication on user experience and adoption of AI-based technology. This extends the literature on biometric authentication and the authentication process of AI during various value chain activities (Ross and Jain, 2004; Mahfouz et al., 2017). In addition, to complement existing studies that focus primarily on non-commercial applications in the healthcare and education sectors (Todorov et al., 2018; de Barcelos Silva et al., 2020), we emphasize the use of voice intelligence in the customer value chain. This contributes to the understanding of user perception toward voice intelligence when consumers evaluate and purchase products and services.

### Managerial implications

This paper provides valuable insights into the user interface design of voice intelligence. First, to incorporate anthropomorphic characteristics into voice intelligence, it is important to address functional intelligence, sincerity, information creativity, and voice tone variation. These elements are associated with user perception and experience. According to our findings, the proper design of these four anthropomorphic characteristics improves the user experience of voice intelligence during the customer journey.

Second, multimodal biometric authentication can significantly improve user perception and motivate users to adopt voice intelligence in the customer value chain. Although our research does not show its association with specific perceived factors, it is evident that the use of multimodal biometric authentication positively influences overall user perception.

Moreover, anthropomorphic characteristics and multimodal biometric authentication complement each other. The benefit of adopting either of them is compelling. When both are utilized, customers may experience a promising interaction with voice intelligence.

Our study also contributes to companies that wish to adopt zero-touch interaction with their customers. We find that individuals between the ages of 35 and 44 and with a high level of education are more likely to adopt voice intelligence in the customer value chain. However, in general, they also have a high concern for privacy. Since they have more knowledge about technology and data collection, they have more awareness of potential risks and privacy issues. High privacy concern negatively influences user adoption. Those who wish to successfully target this segment in the use of voice intelligence must carefully handle customers' personal data involved in the interaction in order to mitigate users' perceived privacy concerns.

### Limitations and future research

As discussed, in our online experiment, subjects did not have the opportunity to directly interact with a voice assistant; we were only able to provide them with a conversation to listen to. Therefore, their perceptions were measured based on indirect user experience. This may have negatively influenced the accuracy of the measurement. In the future, more advanced treatment can be developed to enable a direct verbal dialogue between the subjects and the voice assistance. The creation of a real-life scenario and user experience can enhance the accuracy of the measured user perception.

In addition, the literature on interaction with voice intelligence indicates that environment (such as public and private) is another significant influential factor when users decide on their preferred interaction method (Moorthy and Vu, 2015). Different environments lead to different user perception of social acceptability for interaction with voice intelligence, especially in sharing personal information (Rico and Brewster, 2010; Efthymiou and Halvey, 2016). This environmental factor was not included in our experiment, because it is difficult to manipulate various environment settings online. Future research is encouraged to take the environmental factor into account by letting the user physically interact with voice software in both commercial (e.g., financial institutions) and non-commercial (e.g., healthcare) settings (McKinsey and Company, 2021).

## Data availability statement

The raw data supporting the conclusions of this article will be made available by the authors, without undue reservation.

## Ethics statement

Ethical review and approval was not required for the study on human participants in accordance with the local legislation and institutional requirements. Written informed consent for participation was not required for this study in accordance with the national legislation and the institutional requirements.

## Author contributions

MK conceived the original research idea, carried out the empirical study, performed data analyses, and wrote the manuscript. SY provided comments to improve the research, supervised the project, commented on the study process, and data analyses. Both authors contributed to the article and approved the submitted version.

## Conflict of interest

The authors declare that the research was conducted in the absence of any commercial or financial relationships that could be construed as a potential conflict of interest.

## Publisher's note

All claims expressed in this article are solely those of the authors and do not necessarily represent those of their affiliated organizations, or those of the publisher, the editors and the reviewers. Any product that may be evaluated in this article, or claim that may be made by its manufacturer, is not guaranteed or endorsed by the publisher.

## References

[B1] Aaker J. L. (1997). Dimensions of brand personality. J. Market. Res. 34, 347–356. 10.2307/3151897

[B2] Alepis E. Patsakis C. (2017). Monkey says, monkey does: security and privacy on voice assistants. Digit. Object Ident. 5, 17841–17851. 10.1109/ACCESS.2017.2747626

[B3] Alford S. (2020). GDPR: A Game of Snakes and LADDERS. London: Routledge. 10.4324/9781003004790

[B4] Allen M. (2017). The Sage Encyclopedia of Communication Research Methods, Vols. 1–4. Thousand Oaks, CA: SAGE Publications, Inc. 10.4135/9781483381411

[B5] Amazon (2021). What Is Automatic Speech Recognition? Amazon Developers. Retrieved from: https://developer.amazon.com/en-US/alexa/alexa-skills-kit/asr

[B6] Asfar T. Schmidt M. Kalan M. E. Wu W. Ward K. D.. (2020). Delphi study among international expert panel to develop waterpipe-specific health warning labels. Tobacco Control 29, 159–167. 10.1136/tobaccocontrol-2018-054718 30696784 PMC6663656

[B7] Bajorek J. P. (2019). Voice Recognition Still Has Significant Race and Gender Biases. Harvard Business Review. Retrieved from: https://hbr.org/2019/05/voice-recognition-still-has-significant-race-and-gender-biases

[B8] Bartneck C. Okada M. (2001). “eMuu - an emotional robot,” in Proceedings of the RoboFesta Kansai (Osaka: Technische Universiteit Eindhoven). Retrieved from: https://www.bartneck.de/publications/2001/eMuuAnEmotionalRobot/bartneckRobofesta2001.pdf

[B9] Bhattacharyya D. Ranjan R. Alisherov F. A. Choi M. (2009). Biometric authentication: a review. Int. J. u eServ. Sci. Technol. 2, 13–28. Available online at: https://www.semanticscholar.org/paper/Biometric-Authentication%3A-A-Review-Bhattacharyya-Ranjan/b7bea7d431fb6a55ad39de4933e23c33ab84632a#citing-papers

[B10] Brandt M. (2017). Angenehme Assistenten. Norstat. Retrieved from: https://de.statista.com/infografik/8846/beurteilung-der-stimmen-von-digitalen-sprachassistenten/

[B11] Brewster T. (2017). Voice Squatting' Hack Can Turn Amazon Alexa into a Silent Spy. Forbes. Retrieved from: https://www.forbes.com/sites/thomasbrewster/2018/05/16/amazon-echo-google-home-voice-squatting-attack-exposes-devices/?sh=353e60797533

[B12] Budiu R. (2018). Between-Subjects vs. Within-Subjects Study Design. Nielsen Norman Group. Retrieved from: https://www.nngroup.com/articles/between-within-subjects/

[B13] Cerekovic A. Aran O. Gatica-Perez D. (2017). Rapport with virtual agents: what do human social cues and personality explain? IEEE Trans. Affect. Comput. 8, 382–395. 10.1109/TAFFC.2016.2545650

[B14] Chen Q. Rodgers S. (2006). Development of an instrument to measure web site personality. J. Interact. Advert. 7, 4–46. 10.1080/15252019.2006.1072212420190004

[B15] Chen Q. Roger E. B. Qiu J. (2020). Mapping the artistic brain: common and distinct neural activations associated with musical, drawing, and literary creativity. Nat. Library Med. 41, 3403–3419. 10.1002/hbm.25025 32472741 PMC7375056

[B16] Chérif E. Lemoine J. (2019). Anthropomorphic virtual assistants and the reactions of Internet users: an experiment on the assistant's voice. Recherche et Applications en Marketing 34, 28–47. 10.1177/2051570719829432

[B17] Chitturi R. Raghunathan R. Mahajan V. (2008). Delight by design: the role of hedonic versus utilitarian benefits. J. Market. 72, 48–63. 10.1509/jmkg.72.3.048

[B18] Cho H. Lee J. Chung S. (2010). Optimistic bias about online privacy risks: testing the moderating effects of perceived controllability and prior experience. Comput. Hum. Behav. 26, 987–995. 10.1016/j.chb.2010.02.012

[B19] Choi Y. K. Miracle G. E. Biocca F. (2001). The effects of anthropomorphic agents on advertising effectiveness and the mediating role of presence. J. Interact. Advert. 2, 19–32. 10.1080/15252019.2001.10722055

[B20] Chuah S. H. Rauschnabel P. A. Krey N. Nguyen B. Ramayah T. Lade S. (2016). Wearable technologies: the role of usefulness and visibility insmartwatch adoption. Comput. Hum. Behav. 65, 276–284. 10.1016/j.chb.2016.07.047

[B21] Cohen J. (1988). Statistical Power Analysis for the Behavioral Sciences. Lawrence Earlbaum Associates Publishers. 10.4324/9780203771587

[B22] Cohen J. Cohen P. West S. G. Aiken L. S. (2003). Applied Multiple Regression/Correlation Analysis for the Behavioral Sciences, 3rd Edn. New York, NY: Lawrence Erlbaum Associates Publishers. 10.4324/9780203774441

[B23] Collier G. (1995). Information privacy. Inform. Manage. Comput. Sec. 3, 41–45. 10.1108/09685229510792979

[B24] Condliffe J. (2019). The Week in Tech: Facebook's Privacy Pivot. The New York Times. Retrieved from: https://www.nytimes.com/2019/03/08/technology/facebook-privacy-pivot.html

[B25] Cuofano G. (2021). What Is the Customer Value Chain and Why It Matters. Fourweekmba. Retrieved from: https://fourweekmba.com/customer-value-chain/

[B26] Davis F. D. Bagozzi R. P. Warshaw P. R. (1989). User acceptance of computer technology: a comparison of two theoretical models. Manage. Sci. 35, 982–1003. 10.1287/mnsc.35.8.982

[B27] de Barcelos Silva A. Gomes M. M. da Costa C. A. da Rosa Righi R. Barbosa J. L. V. Pessin G.. (2020). Intelligence personal assistants: a systematic literature review. Expert Syst. Appl. 147, 113–193. 10.1016/j.eswa.2020.113193

[B28] de Boer J. Drukker J. W. (2011). High Tech Human Touch. Enschede: Lecturis Printing Company. 10.3990/1.9789036532730

[B29] DeCastellarnau A. (2018). A classification of response scale characteristics that affect data quality: a literature review. Qual. Quant. 52, 1523–1559. 10.1007/s11135-017-0533-4 29937582 PMC5993837

[B30] Delić V. Sečujski M. Sedlar N. V. Mišković D. Mak R. Bojanić M. (2014). How speech technologies can help people with disabilities. Int. Conf. Speech Comput. 8773, 243–250. 10.1007/978-3-319-11581-8_30

[B31] Dimov D. (2015). Security Vulnerabilities of Voice Recognition Technologies. Infosec. Retrieved from: https://resources.infosecinstitute.com/topic/security-vulnerabilities-of-voice-recognition-technologies/

[B32] Duffy B. R. (2003). Anthropomorphism and the social robot. Robots Auton. Syst. 42, 177–190. 10.1016/S0921-8890(02)00374-3

[B33] Edwards C. Edwards A. Stoll B. Lin X. Massey N. (2019). Evaluations of an artificial intelligence instructor's voice: social identity theory in human-robot interactions. Comput. Hum. Behav. 90, 357–362. 10.1016/j.chb.2018.08.027

[B34] Efthymiou C. Halvey M. (2016). Evaluating the social acceptability of voice based smartwatch search. Infm. Retrieval Technol. 9994, 267–278. 10.1007/978-3-319-48051-0_20

[B35] El-Abed M. Giot R. Hemery B. Rosenberger C. (2010). “A study of users' acceptance and satisfaction of biometric systems,” in 44th Annual 2010 IEEE International Carnahan Conference on Security Technology (San Jose, CA). 170–178. 10.1109/CCST.2010.5678678

[B36] Exline J. J. Geyer A. L. (2004). Perceptions of humility: a preliminary study. Self Identity 3, 95–114. 10.1080/13576500342000077 21526606

[B37] Ezrachi A. Stucke M. E. (2017). Artificial Intelligence and Collusion: When Computer Inhibit Competition. Illinois Law Review. Retrieved from: https://www.illinoislawreview.org/wp-content/uploads/2017/10/Ezrachi-Stucke.pdf

[B38] Field A. Hole G. (2003). How to Design and Report Experiments. Sussex: SAGE Publications.

[B39] Fornell C. Larcker D. F. (1981). Evaluating structural equation models with unobservable variables and measurement error. J. Market. Res. 18, 39–50. 10.2307/3151312

[B40] Fritz M. S. Taylor A. B. Mackinnon D. P. (2012). Explanation of two anomalous results in statistical mediation analysis. Multivariat. Behav. Res. 47, 61–87. 10.1080/00273171.2012.640596 24049213 PMC3773882

[B41] Gardner D. G. Dukes R. L. Discenza R. (1993). Computer use, self-confidence, and attitudes: a causal analysis. Comput. Hum. Behav. 9, 427–440. 10.1016/0747-5632(93)90033-O

[B42] Gourlay A. Mshana G. Birdthistle I. Bulugu G. Zaba B. Urassa M. (2014). Using vignettes in qualitative research to explore barriers and facilitating factors to the uptake of prevention of mother-to-child transmission services in rural Tanzania: a critical analysis. BMC Med. Res. Methodol. 14, 21. 10.1186/1471-2288-14-21 24512206 PMC3922981

[B43] Graeff T. R. Harmon S. (2002). Collecting and using personal data: consumers' awareness and concerns. J. Cons. Market. 19, 302–318. 10.1108/07363760210433627

[B44] Guzman A. L. (2018). Voices in and of the machine: source orientation toward mobile virtual assistants. Comput. Hum. Behav. 90, 343–350. 10.1016/j.chb.2018.08.009

[B45] Hackbarth G. Grover V. Yi M. Y. (2003). Computer playfulness and anxiety: positive and negative mediators of the system experience effect on perceived ease of use. Inform. Manage. 40, 221–232. 10.1016/S0378-7206(02)00006-X

[B46] Haerling Adamson K. Prion S. (2020). Two-by-two factorial design. Clin. Simul. Nurs. 49, 90–91. 10.1016/j.ecns.2020.06.004 33024457 PMC7529390

[B47] Hair J. F. Ringle C. M. Sartedt M. (2011). PLS-SEM: indeed a silver bullet. J. Market. Theory Pract. 19, 139–152. 10.2753/MTP1069-6679190202

[B48] Hair J. F. Risher J. J. Sarstedt M. Ringle C. M. (2019). When to use and how to report the results of PLS-SEM. Eur. Bus. Rev. 31, 2–24. 10.1108/EBR-11-2018-0203

[B49] Hamid M. R. Sami W. Sidek M. H. M. (2017). Discriminant validity assessment: use of Fornell and Larcker criterion versus HTMT criterion. J. Phys. Conf. Ser. 890:012163. 10.1088/1742-6596/890/1/012163

[B50] Hanzo L. Clare F. Somerville A. Woodard J. (2000). Voice Compression and Communications. Ann Arbor, MI: John Wiley and Sons.

[B51] Hassenzahl M. Tractinsky N. (2011). User experience - a research agenda. Behav. Inform. Technol. 25, 91–97. 10.1080/01449290500330331

[B52] Hazen T. J Weinstein E Heisele B Park A Ming J. (2007). “Multimodal face and speaker identification for mobile devices,” in Face Biometrics for Personal Identification, eds R. I. Hammoud, B. R. Abidi, and M. A. Abidi (Berlin: Springer), 123–138. 10.1007/978-3-540-49346-4_9

[B53] Hazen T. J. Weinstein E. Kabir R. Park A. Heisele B. (2003). “Multi-modal face and speaker identification on a handheld device,” in Proceedings of the Workshop on Multimodal User Authentication (Santa Barbara, CA), 113–120. Available online at: https://citeseerx.ist.psu.edu/viewdoc/citations?doi=10.1.1.2.9548

[B54] Hearling K. Prion S. (2020). Two-by-two factorial design. Clin. Simulat. Nurs. 49, 90–91. Available online at: https://www.nursingsimulation.org/action/showPdf?pii=S1876-1399%2820%2930052-9 10.1016/j.ecns.2020.06.004 PMC7529390 33024457

[B55] Hirschberg J. Manning C. D. (2015). Advances in natural language processing. Science 349, 261–266. 10.1126/science.aaa8685 26185244

[B56] Hoy M. D. (2018). Alexa, Siri, Cortana, and more: an introduction to voice assistants. Med. Ref. Serv. Q. 37, 81–88. 10.1080/02763869.2018.1404391 29327988

[B57] ITU (2019). Measuring Digital Development: Facts and Figures. ITUPublications. Retrieved from: https://www.itu.int/en/mediacentre/Documents/MediaRelations/ITU%20Facts%20and%20Figures%202019%20-%20Embargoed%205%20November%201200%20CET.pdf

[B58] Jadczyk T. Wojakowski W. Tendera M. Henry T. Egnaczyk G. Shreenivas S. (2021). Artificial intelligence can improve patient management at the time of a pandemic: the role of voice technology. J. Med. Internet Res. 23:e22959. 10.2196/22959 33999834 PMC8153030

[B59] Jain A. K. Ross A. Prabhakar S. (2004). An introduction to biometric recognition. IEEE Trans. on Circuits and Systems for Video Technology 14, 4–20. 10.1109/TCSVT.2003.818349

[B60] Janssen A. Passlick J. Cardona D. R Breitner M, H. (2020). Virtual assistance in any context: a taxonomy of design elements for domain-specific chatbots. Bus. Inform. Syst. Eng. 62, 211–225. 10.1007/s12599-020-00644-1

[B61] Johnson D. C. Bauer B. C. Singh N. (2020). Exploring flow in the mobile interface context. J. Retail. Cons. Serv. 53:101744. 10.1016/j.jretconser.2019.01.013

[B62] Johnstone T. Scherer K. (2000). “Vocal communication of emotion,” in The Handbook of Emotions, eds M. Lewis and J. Haviland (New York, NY: Guilford), 220–232.

[B63] Jokinen J. P. P. (2015). Emotional user experience: traits, events, and states. Int. J. Hum. Comput. Stud. 76, 67–77. 10.1016/j.ijhcs.2014.12.006

[B64] Julious S. A. (2005). Sample size of 12 per group rule of thumb for a pilot study. Pharmaceut. Stat. 4, 287–291. 10.1002/pst.185

[B65] Kemp D. (2019). Using Smart Speakers to Engage With Your Customers. Harvard Business Review. Retrieved from: https://hbr.org/2019/05/using-smart-speakers-to-engage-with-your-customers

[B66] Kim S. Gajos M. K. Z. Muller M. Grosz B. J. (2016). Acceptance of mobile technology by older adults: a preliminary study. MobileHCI 16, 147–157. 10.1145/2935334.2935380

[B67] Kinsella B. (2019). Smart Speaker Consumer Adoption. Southport, NC: Voicebot.

[B68] Kinsella B. (2020). Amazon Alexa Skill Growth Has Slowed Further in 2020. Voicebot. Retrieved from: https://voicebot.ai/2020/10/25/amazon-alexa-skill-growth-has-slowed-further-in-2020/#:~:text=In%20all%20of%202018%2C%20new,growth%20rate%20tells%20the%20story

[B69] Klaus P. Zaichkowsky L. J. (2020). The convenience of shopping via voice AI: introducing AIDM. J. Retail. Customer Serv. 65. 10.1016/j.jretconser.2021.102490

[B70] Koopmans L. Bernaards C. M. Hildebrandt V. H. de Vet H. C. van der Beek A. J. (2014). Construct validity of the individual work performance questionnaire. J. Occup. Environ. Med. 56, 331–337. 10.1097/JOM.0000000000000113 24561507

[B71] Landheer J. A. van den Wittenboer G. (2015). Unbalanced 2 x 2 factorial designs and the interaction effect: a troublesome combination. PLoS ONE 10, e0121412. 10.1371/journal.pone.0121412 25807514 PMC4373880

[B72] Lazarus R. S. (2001). “Relational meaning and discrete emotions,” in Appraisal Processes in Emotion: Theory, Methods, Research, eds K. R. Scherer, A. Schorr, and T. Johnstone (Oxford: Oxford University Press), 37–67.

[B73] Lazarus R. S. Averill J. R. Opton E. M. Jr. (1970). “Toward a cognitive theory of emotion,” in Third International Symposium on Feelings and Emotions, ed M. Arnold (New York, NY: Academic Press).

[B74] Lee H. Yang H. (2019). Understanding user behavior of virtual personal assistant devices. Inform. Syst. eBus. Manage. 17, 65–87. 10.1007/s10257-018-0375-1

[B75] Li M. Suh A. (2021). “Machinelike or humanlike? A literature review of anthropomorphism in AI-enabled technology,” in 54th Hawaii International Conference on System Sciences (Manoa, HI), 4053–4062. 10.24251/HICSS.2021.493

[B76] Liu J. Kummerow C. D. Elsaesser G. S. (2017). Identifying and analyzing uncertainty structures in the TRMM Microwave Imager precipitation product. Int. J. Remote Sens. 38, 23–42. 10.1080/01431161.2016.1259676

[B77] Liu S. (2021). Global Voice Recognition Market Size 2019 and 2025. Statista. Retrieved from: https://www.statista.com/statistics/1133875/global-voice-recognition-market-size/#statisticContainer

[B78] Mahfouz A. Mahmoud T. M. Eldin A. S. (2017). A survey on behavioral biometric authentication on smartphones. J. Inform. Sec. Appl. 37, 28–37. 10.1016/j.jisa.2017.10.002

[B79] Mahlke S. Minge M. (2008). Consideration of multiple components of emotions in human-technology interaction. Affect Emot. Hum. Comput. Interact. 4868, 51–62. 10.1007/978-3-540-85099-1_524721040

[B80] Max Planck Institute for Psycholinguistics (2019). Speech Recognition Technology Is Not a Solution for Poor Readers. Sciencedaily. Retrieved from: https://www.sciencedaily.com/releases/2019/05/190513112229.htm

[B81] McKinsey Company (2021). The Next Normal: The Recovery Will Be Digital. McKinsey and Company. Retrieved from: https://www.mckinsey.com/~/media/mckinsey/business%20functions/mckinsey%20digital/our%20insights/how%20six%20companies%20are%20using%20technology%20and%20data%20to%20transform%20themselves/the-next-normal-the-recovery-will-be-digital.pdf

[B82] McLean G. Osei-Frimpong K. (2019). Hey Alexa … examine the variables influencing the use of artificial intelligent in-home voice assistants. Comput. Hum. Behav. 99, 28–37. 10.1016/j.chb.2019.05.009

[B83] Melián-González S. Gutiérrez-Tãno D. Bulchand-Gidumal J. (2021). Predicting the intentions to use chatbots for travel and tourism. Curr. Issues Tourism 24, 192–210. 10.1080/13683500.2019.1706457

[B84] Moller A. C. Deci E. L. Ryan R. M. (2006). Choice and ego-depletion: the moderating role of autonomy. Pers. Soc. Psychol. Bull. 30, 1024–1036. 10.1177/0146167206288008 16861307

[B85] Moon Y. (2000). Intimate exchanges: using computers to elicit self-disclosure from consumers. J. Cons. Res. 26, 323–339. 10.1086/209566

[B86] Moore G. (1991). Crossing the Chasm: Marketing and Selling High-Tech Products to Mainstream Customers. Harper Business. Retrieved from: http://soloway.pbworks.com/w/file/fetch/46715502/Crossing-The-Chasm.pdf

[B87] Moorthy A. E. Vu L. K. (2015). Privacy concerns for use of voice activated personal assistant in the public space. Int. J. Hum. Comput. Interact. 31, 307–335. 10.1080/10447318.2014.986642

[B88] Moran K. (2016). The Four Dimensions of Tone of Voice. Nielsen Group. Retrieved from: https://www.nngroup.com/articles/tone-of-voice-dimensions/

[B89] Moussawi S. Koufaris M. Benbunan-Fich R. (2020). How perceptions of intelligence and anthropomorphism affect adoption of personal intelligent agents. Electron Markets 31, 343–364. 10.1007/s12525-020-00411-w

[B90] Nass C. Brave S. (2005). Wired for Speech: How Voice Activates and Advances the Human-Computer Relationship. MIT Press. Retrieved from: https://www.researchgate.net/publication/220355274_Wired_for_Speech_How_Voice_Activates_and_Advances_the_Human-Computer_Relationship

[B91] Nass C. Lee K, M. (2001). Does computer-synthesized speech manifest personality? J. Exper. Psychol. 7, 171–181. 10.1037//1076-898X.7.3.171 11676096

[B92] Oberoi A. (2019). The Rise of Voice User Interface (VUI). Daffodil. Retrieved from: https://insights.daffodilsw.com/blog/the-rise-of-voice-user-interface-vui

[B93] Oloyede M. O. Hancke G. P. (2016). Unimodal and multimodal biometric sensing systems: a review. IEEE Access 4, 7532–7555. 10.1109/ACCESS.2016.2614720

[B94] Pederson T. Jalaliniya S. (2015). “An egocentric approach towards ubiquitous multimodal interaction,” in ACM International Joint Conference on Pervasive and Ubiquitous Computing and Proceedings of the 2015 ACM International Symposium on Wearable Computers (Copenhagen), 927–932. 10.1145/2800835.2806202

[B95] Pemberton C. (2018). Is Your Product Development Ready for Zero-Touch User Interfaces? Gartner. Retrieved from: https://www.gartner.com/smarterwithgartner/is-your-product-development-ready-for-zero-touch-user-interfaces/

[B96] Peter J. P. (1981). Construct validity: a review of basic issues and marketing practices. J. Market. Res. 18, 133–145. 10.1177/002224378101800201

[B97] Pieraccini R. (2012). The Voice in the Machine: Building Computers That Understand Speech. Cambridge, MA: MIT Press. 222–230. 10.1080/10447318.2012.715051

[B98] Piffer D. (2012). Can creativity be measured? An attempt to clarify the notion of creativity and general directions for future research. Think. Skills Creat. 7, 258–264. 10.1016/j.tsc.2012.04.009

[B99] Pitardi V. Marriott H. R. (2021). Alexa, she's not human but… Unveiling the drivers of consumers' trust in voice-based artificial intelligence. Psychol. Market. 38, 626–642. 10.1002/mar.21457

[B100] Porcheron M. Fischer J. E. Reeves S. Sharples S. (2018). “Voice interfaces in everyday life,” in Proceedings of the 2018 CHI Conference on Human Factors in Computing Systems (Nottingham), 1–12. 10.1145/3173574.3174214

[B101] Poushneh A. (2021). Humanizing voice assistant: the impact of voice assistant personality on consumers' attitudes and behaviors. J. Retail. Cons. Serv. 58:102283. 10.1016/j.jretconser.2020.102283

[B102] PWC (2018). Consumer Intelligence Series: Prepare for the Voice Revolution. Retrieved from: https://www.pwc.com/us/en/services/consulting/library/consumer-intelligence-series/voice-assistants.html

[B103] Qiu H. Li M. Shu B. Bai B. (2020). Enhancing hospitality experience with service robots: the mediating role of rapport building. J. Hosp. Market. Manage. 29, 247–268. 10.1080/19368623.2019.1645073

[B104] Quatieri T. F. (2002). Speech Signal Processing – Principles and Practice. Lexington, MA: Prentice Hall Signal Processing Series.

[B105] Rashid R. A. Mahalin N. H. Sarijari M. A. Aziz A. A. A. (2008). “Security system using biometric technology: design and implementation of voice recognition system (VRS),” in International Conference on Computer and Communication Engineering (Kuala Lumpur), 898–902. 10.1109/ICCCE.2008.4580735

[B106] Rauschnabel P. A. He J. Ro Y. K. (2018). Antecedents to the adoption of augmented reality smart glasses: a closerlook at privacy risks. J. Bus. Res. 92, 374–384. 10.1016/j.jbusres.2018.08.008

[B107] Rico J. Brewster S. (2010). “Usable gestures for mobile interfaces: evaluating social acceptability,” in Proceedings of the SIGCHI Conference on Human Factors in Computing Systems (Glasgow), 887–896. 10.1145/1753326.1753458

[B108] Rogers E. M. (1995). Diffusion of Innovations, 4th Edn. Vienna: The Free Press.

[B109] Ross A. Jain A. K. (2004). Multimodal Biometrics: An Overview. EUSIPCO, 1221–1224 Retrieved from: http://biometrics.cse.msu.edu/Publications/Multibiometrics/RossJain_MultimodalOverview_EUSIPCO04.pdf

[B110] Ryu R. Yeom S. Kim S. Herbert D. (2021). Continuous multimodal biometric authentication schemes: a systematic review. Inst. Electric. Electron. Eng. 9, 34541–34557. 10.1109/ACCESS.2021.3061589

[B111] Rzepka C. Berger B. (2018). “User interaction with AI-enabled systems: a systematic review of IS research,” in Thirty Ninth International Conference on Information Systems. Retrieved from: https://aisel.aisnet.org/icis2018/general/Presentations/7/

[B112] Scherer K. R. (2009). The dynamic architecture of emotion: evidence for the component process model. Cogn. Emot. 23, 1307–1351. 10.1080/02699930902928969

[B113] Schwär H. Moynihan Q. (2020). Companies Like Amazon May Give Devices Like Alexa Female Voices to Make Them Seem 'Caring'. Business Insider Deutschland. Retrieved from: https://www.businessinsider.com/theres-psychological-reason-why-amazon-gave-alexa-a-female-voice-2018-9?international=trueandr=USandIR=T

[B114] Sethi A. Adhikari B. (2010). Impact of communicating ‘Vision' on organizational communication effectiveness. Int. J. Market. Bus. Commun. 1, 43–48. Available online at: http://www.publishingindia.com/GetBrochure.aspx?query=UERGQnJvY2h1cmVzfC8xMjYwLnBkZnwvMTI2MC5wZGY=

[B115] Sezgin E. Huang Y. Ramtekkar U. Lin S. (2020). Readiness for voice assistants to support healthcare delivery during a health crisis and pandemic. Digit. Med. 3, 122. 10.1038/s41746-020-00332-0 33015374 PMC7494948

[B116] Shugan S. M. (1980). The cost of thinking. J. Cons. Res. 7, 99–111. 10.1086/208799

[B117] Singh S. (2018). Sampling Techniques. Towards Data Science. Retrieved from: https://towardsdatascience.com/sampling-techniques-a4e34111d3808

[B118] Stets J. E. Burke P. J. (1996). Gender, control, and interaction. Soc. Psychol. Q. 59, 193–220.

[B119] Suresh K. P. (2011). An overview of randomization techniques: an unbiased assessment of outcome in clinical research. J. Hum. Reproduct. Sci. 4, 8–11. 10.4103/0974-1208.82352 21772732 PMC3136079

[B120] Szumski O. (2020). Technological trust from the perspective of digital payment. Proc. Comput. Sci. 176, 3545–3554. 10.1016/j.procs.2020.09.032

[B121] Thabane L. Ma J. Chu R. Cheng J. Ismaila A. Rios L.. (2010). A tutorial on pilot studies: the what, why and how. BMC Med. Res. Methodol. 10, 1. 10.1186/1471-2288-10-1 20053272 PMC2824145

[B122] Thakkar D. (2021). Unimodal Biometrics vs. Multimodal Biometrics. Bayometric. Retrieved from: https://www.bayometric.com/unimodal-vs-multimodal/

[B123] Thüring M. Mahlke S. (2007). Usability, aesthetics and emotions in human-technology interaction. Psychol. Press 42, 253–264. 10.1080/00207590701396674

[B124] Toader D. Boca G. Toader R. Macelaru M. Toader C. Ighian D.. (2020). The effect of social presence and chatbot errors on trust. Sustainability 12, 256. 10.3390/su12010256

[B125] Todorov J. Valkanov V. Stoyanov S. Daskalov B. Popchev I. Orozova D. (2018). Personal assistants in a virtual education space. Pract. Issu. Intell. Innov. 140, 131–153. 10.1007/978-3-319-78437-3_6

[B126] van Biljon J. Kotzé P. (2004). “Modelling the factors that influence mobile phone adoption,” in SAICSIT'07: Proceedings of the 2018 Annual Research Conference of the South Africa Institute of Computer Scientists and Information Technologies on IT Research in Developing Countries (Glasgow), 152–161. 10.1145/1292491.1292509

[B127] van Biljon J. Renaud K. (2008). “Predicting technology acceptance and adoption by the elderly: a qualitative study,” in SAICSIT'08: Proceedings of the 2018 Annual Research Conference of the South Africa Institute of Computer Scientists and Information Technologies on IT Research in Developing Countries: Riding the Wave of Technology (Glasgow), 210–219. 10.1145/1456659.1456684

[B128] Verhagen T. van Nes J. Feldberg F. van Dolen W. (2014). Virtual customer service agents: using social presence and personalization to shape online service encounters. J. Comput. Mediat. Commun. 19, 529–545. 10.1111/jcc4.12066

[B129] Wagner K. Nimmermann F. Schramm-Klein H. (2019). “Is it human? The role of anthropomorphism as a driver for the successful acceptance of digital voice assistants,” in Proceedings of the 52nd Hawaii International Conference on System Sciences (Siegen). Retrieved from: http://128.171.57.22/bitstream/10125/59579/1/0139.pdf

[B130] Wang E. S. T. (2015). Different effects of utilitarian and hedonic benefits of retail food packaging on perceived product quality and purchase intention. J. Food Products Market. 23, 239–250. 10.1080/10454446.2014.885867

[B131] Waytz A. Heafner J. Epley N. (2014). The mind in the machine: anthropomorphism increases trust in an autonomous vehicle. J. Exp. Soc. Psychol. 52, 113–117. 10.1016/j.jesp.2014.01.005

[B132] Westcott K. Loucks J. Littmann D. Wilson P. Srivastava S. Ciampa. (2018). Build It and They Will Embrace It Consumers Are Preparing for 5G Connectivity in the Home and on the Go. The Deloitte Centre for Technology, Media and Telecommunication. Retrieved from: https://www2.deloitte.com/content/dam/insights/us/articles/6457_Mobile-trends-survey/DI_Build-it-and-they-will-embrace-it.pdf

[B133] Wong Y. K. Hsu C. J. (2008). A confidence-based framework for business to consumer (B2C) mobile commerce adoption. Pers. Ubiquit. Comput. 12, 77–84. 10.1007/s00779-006-0120-5

[B134] Wu Z. Evans N. Kinnunen T. Yamagishi J. Alegre F. Li H. (2015). Spoofing and countermeasures for speaker verification: a survey. Speech Commun. 66, 130–153. 10.1016/j.specom.2014.10.005

[B135] Zeng L. Proctor R. Salvendy G. (2011). Can traditional divergent thinking tests be trusted in measuring and predicting real-world creativity? Creat. Res. J. 23, 24–37. 10.1080/10400419.2011.545713

[B136] Zhang N. Mi X. Feng X. Wang X. Tian Y. Qian F. (2019). “Dangerous skills: understanding and mitigating security risks of voice controlled third party functions on virtual personal assistant systems,” in 2019 IEEE Symposium on Security and Privacy (SP) (San Francisco, CA), 1381–1396. 10.1109/SP.2019.00016

[B137] Zhang Y. Chen J. Q. Wen K. W. (2002). Characteristics of internet users and their privacy concerns. J. Internet Comm. 1, 1–16. 10.1300/J179v01n02_0135235434

[B138] Zhu Q. Wang T. Jia Y. (2007). “Second life: a new platform for education,” in 2007 First IEEE International Symposium on Information Technologies and Applications in Education (Kunming), 201–204. 10.1109/ISITAE.2007.4409270

[B139] Zhu Z. Branzoi V. Wolverton M. Murray G. Vitovitch N. Yarnall L. (2014). “AR-mentor: augmented reality based mentoring system,” in 2014 IEEE International Symposium on Mixed and Augmented Reality (ISMAR) (Munich), 17–22. 10.1109/ISMAR.2014.6948404

