# Supplementary material for: The effects of anthropomorphism and multimodal biometric authentication on the user experience of voice intelligence
Source: Front Artif Intell. 2022 Aug 17;5:831046. doi: 10.3389/frai.2022.831046 (PMC9428311; doi:10.3389/frai.2022.831046)
Supplement: Supplementary file 1 [file Table_1.DOCX]

**Appendix I. Treatment transcription**

| Manipulation | Color |
| --- | --- |
| *Biometric authentication* |  |
| *Functional intelligence* |  |
| *Sincerity* |  |
| *Information creativity* |  |
| *Voice intonation: causal* |  |
| *Voice intonation: enthusiasts* |  |
| *Voice intonation: formal* |  |

### Conversation #1: NANT | NBA (T1)

Activity #1: (Daily activities)

**The weather looks nice today. You ask Iris to get more information.**

**User:** “The weather looks lovely today: Iris, what is the weather?”

**Iris:** “Currently, in Rotterdam, it is 8 degrees Celsius with clear skies and sun. Today, You can expect intermediated clouds with a chance of rain with a high of 9 degrees and a low of 0 degrees”.

Activity #2: (Daily activities)

**It is time for breakfast. You ask Iris to boil some eggs.**

**User:** “Let’s boil some eggs for breakfast: Iris, set an 8-minute timer and name it eggs".

**Iris:** “Set time eggs for 8 minutes starting now.”

Activity #3: (Daily activities)

**While cooking, you realize that there is almost out of milk. You ask Iris to add this product to your grocery list.**

**User**: “Hmm, we are almost out of milk: Iris, add half a liter of fat free milk to my shopping list”

**Iris:** “The suggested milk is: half a liter of Campina fat free milk for 2 dollar and 59 cents, would You like to add this to Your list?”

**User:** “Yes.”

**Iris:** “Added half a liter of Campina fat free milk to the grocery list.”

Activity #4: (Daily activities)

**You want to purchase the grocery list while using Iris.**

**User:** “It is time to do some groceries: Iris, which items are on my grocery list?”

**Iris**: “You have four items on Your grocery list:

- Half a liter of Campina fat free milk for 2 dollar and 59 cents.
- 2 rolls of Nalys kitchen paper for 3 dollar and 85 cents.
- 1L of Bertolli olive oil for 9 dollar and 39 cents.
- 23 sun dishwasher cubes for 4 dollar and 79 cents.

The total of all products is 20 dollar 62 cents. Would You like to buy this list?”

**User**: “Yes”

**Iris**: “Ordered all products from the list. The groceries will be delivered on Friday at 1’o clock in the afternoon.”

Activity #5: (Relational exchanges)

**You want to transfer some money between banking accounts while using Iris.**

**User**: “I want to transfer some money to my savings account: Iris, check the balance of my checking account.”

**Iris**: “Your checking account available balance is: 100 dollar and 56 cents.”

**User:** “Iris, ask Bank of America to transfer 45 dollars from my checking account to my savings account.”

**Iris**: “Please say Your PIN to perform this transaction.”

**User**: “452957”

**Iris**: “PIN correct, your transfer from checking account to savings account of 45 dollar has been successful.”

Activity #6: (Relational exchanges)

**You want to perform a banking transaction while using Iris.**

**User:** “I think I owe Peter some money: Iris, check the balance of my checking account.”

**Iris**: “Your checking account available balance is: 55 dollar and 56 cents.”

**User** “Iris, ask Bank of America to send 50 dollar to Peter White.”

**Iris**: “Please say Your PIN-code to perform this transaction.”

**User:** “452957”

**Iris**: “Your pin is correct. I found Peter White in the contact list. Your transfer from checking account to Peter White of 50 dollar has been successful.”

### Conversation #2: NANT | BA (T2)

Activity #1: (daily activities)

**The weather looks nice today. You ask Iris to get more information.**

**User:** “The weather looks lovely today: Iris, what is the weather?”

**Iris**: “Currently, in Rotterdam, it is 8 degrees Celsius with clear skies and sun. Today, You can expect intermediated clouds with a chance of rain with a high of 9 degrees and a low of 0 degrees”.

Activity #2: (Daily activities)

**It is time for breakfast. You ask Iris to boil some eggs.**

**User:** “Let’s boiler some eggs for breakfast: Iris, set an 8-minute timer and name it eggs".

**Iris:** “Set time eggs for 8 minutes starting now.”

Activity #3: (Daily activities)

**While cooking, you realize that there is almost out of milk. You ask Iris to add this product to your grocery list.**

**User**: “Hmm, we are almost out of milk: **Iris, add 0.5L of fat free milk to my shopping list**”

**Iris:** “The suggested milk is: half a liter of Campina fat free milk for 2 dollar and 59 cents, would You like to add this to Your list?”

**User:** “Yes.”

**Iris:** “Added half a liter of Campina fat free milk to the grocery list.”

Activity #4: (Relational exchanges)

**You want to purchase the grocery list while using Iris.**

**User:** “It is time to do some groceries: **Iris, which items are on my grocery list?”**

**Iris**: “You have four items on Your grocery list:

- Half a liter of Campina fat free milk for 2 dollar and 59 cents.
- 2 rolls of Nalys kitchen paper for 3 dollar and 85 cents.
- 1L of Bertolli olive oil for 9 dollar and 39 cents.
- 23 sun dishwasher cubes for 4 dollar and 79 cents.

The total of all products is 20 dollar 62 cents. Would You like to buy this list?”

**User:** “Yes”

**Iris:** ‘Order request confirmed, please scan Your face to authorize Your purchase’

**User:** *Scans *

**Iris**: “Thank You, the face identification for this order request is confirmed. All products from the list are ordered. The groceries will be delivered on Friday at 1’o clock in the afternoon.”

Activity #5: (Relational exchanges)

**You want to transfer some money between banking accounts while using Iris.**

**User**: “I want to transfer some money to my savings account: **Iris, check the balance of my checking account.”**

**Iris**: “Your checking account available balance is: 100 dollar and 56 cents.”

**User:** “Iris, ask Bank of America to transfer 45 dollar form my checking account to my savings account.”

**Iris:** “Transfer request confirmed. Please scan your face to authorize this transfer.”

**User:** *Scans *

**Iris**: “Thank You, the face identification for this money transfer is confirmed. Please say Your PIN to perform this transaction.”

**User** “452957”

**Iris**: “PIN correct, Your transfer from checking account to savings account of 45 dollar has been successful.”

Activity #6: (Relational exchanges)

**You want to perform a banking transaction while using Iris.**

**User:** “I owe Peter some money: **Iris, check the balance of my checking account.”**

**Iris**: “Your checking account available balance is: 55 dollar and 56 cents.”

**User**: “Iris, ask Bank of America to send 50 dollar to Peter White.”

**Iris:** “Transaction request confirmed. Please scan Your face to authorize this transaction.”

**User:** *Scans *

**Iris**: “Thank You, the face identification is confirmed.

**Iris**: “Please say Your PIN-code to perform this transaction.”

**User**: “452957”

**Iris**: “Found Peter White in the contact list. Your transfer from checking account to Peter White of 50 dollar has been successful.”

### Conversation #3: ANT | NBA (T3)

Activity #1: (Daily activities)

**The weather looks nice today. You ask Iris to get more information.**

User: “Oh, the weather looks lovely today!” **“Iris, what is the weather?”**

Iris: “Hey. I’m happy to help You today! The weather looks very inviting. Currently, in New York, it is 8 degrees Celsius with clear skies and sun. Today, you can expect intermediated clouds with a chance of rain with a high of 9 degrees and a low of 0 degrees”. Would You like to receive an advise on what to wear today outside?

User: “Yes”

Iris: Okay. Looking back at previous weather forecasts, I would advise to wear a warm jacket outside. Enjoy!

Activity #2: (Daily activities)

**It is time for breakfast. You ask Iris to boil some eggs.**

**User:** “Lets boiled some eggs for breakfast.” **“Iris, set an 8-minute timer and name it eggs".**

**Iris:** “Good. Set time eggs for 8 minutes starting now. This will make your eggs hard boiled. Do you want me to play some soothing morning music?”

**User:** “No, thanks”

**Iris:** “Okay, enjoy Your day!”

Activity #3: (Daily activities)

**While cooking, you realize that there is almost out of milk. You ask Iris to add this product to your grocery list.**

**User:** “Iris, add 0.5L of fat free milk to my shopping list”

**Iris:** “Hey. Looking back at previous milk purchases, I would like to suggest this product: half a liter of Campina fat free milk for 2 dollar 59 cents, do You like to add this to Your list?”

**User:** “Yes.”

**Iris:** “lovely! Added half a liter of Campina fat free milk to the grocery list.” This product is discounted today, should I order it directly?

**User:** “No, thanks”

**Iris:** “Okay, I only added this product to Your list. enjoy Your day!”

Activity #4: (Relational exchanges)

**You want to purchase the grocery list while using Iris.**

**User:** “Iris, which items are on my grocery list?”

**Iris**: “You have four items on Your grocery list:

- Half a liter of Campina fat free milk for 2 dollar and 59 cents.
- 2 rolls of Nalys kitchen paper for 3 dollar and 85 cents.
- 1L of Bertolli olive oil for 9 dollar and 39 cents.
- 23 sun dishwasher cubes for 4 dollar and 79 cents.

The total of all products is 20 dollar 62 cents. Would You like to buy this list?”

**User:** “Yes”

**Iris**: “Thank You for Your confirmation. Ordered all products from the list. The groceries will be delivered on Friday at 1’o clock in the afternoon.”

Activity #5: (Relational exchanges)

**You want to transfer some money between banking accounts while using Iris.**

**User:** “Iris, check the balance of my checking account.”

**Iris**: “Your checking account available balance is: 100 dollar and 56 cents.”

**User:** “Iris, ask Bank of America to transfer 45 dollar form my checking account to my savings account.”

**Iris**: “You are about the execute a transfer transaction. Please say Your PIN to perform this transaction.”

**User**: “452957”

**Iris**: “Thank You for Your input. The given PIN correct, Your transfer from checking account to savings account of 45 dollar has been successful.”

Activity #6: (Relational exchanges)

**You want to perform a banking transaction while using Iris.**

**User:** “Iris, check the balance of my checking account.”

**Iris**: “Your checking account available balance is: 55 dollar and 56 cents.”

**User**: “Iris, ask Bank of America to send 50 dollar to Peter White.”

**Iris**: “You are about the execute a transfer transaction. Please, say Your PIN-code to perform this transaction.”

**User:** “452957”

**Iris**: “Thank You for Your input. The given PIN correct. I Found Peter White in the contact list. Your transfer from checking account to Peter White of 50 dollar has been successful.”

### Conversation #4: ANT | BA (T4)

Activity #1: (Daily activities)

**The weather looks nice today. You ask Iris to get more information.**

User: “Oh, the weather looks lovely today!” **“Iris, what is the weather?”**

Iris: “Hey. I’m happy to help You today! The weather looks very inviting. Currently, in New York, it is 8 degrees Celsius with clear skies and sun. Today, you can expect intermediated clouds with a chance of rain with a high of 9 degrees and a low of 0 degrees”. Would You like to receive an advise on what to wear today outside?

User: “Yes”

Iris: Okay. Looking back at previous weather forecasts, I would advise to wear a warm jacket outside. Enjoy!

Activity #2: (Daily activities)

**It is time for breakfast. You ask Iris to boil some eggs.**

**User:** “Lets boiled some eggs for breakfast.” **“Iris, set an 8-minute timer and name it eggs".**

**Iris:** “Good. Set time eggs for 8 minutes starting now. This will make your eggs hard boiled. Do you want me to play some soothing morning music?”

**User:** “No, thanks”

**Iris:** “Okay, enjoy Your day!”

Activity #3: (Daily activities)

**While cooking, you realize that there is almost out of milk. You ask Iris to add this product to your grocery list.**

**User:** “Iris, add 0.5L of fat free milk to my shopping list”

**Iris:** “Hey. Looking back at previous milk purchases, I would like to suggest this product: half a liter of Campina fat free milk for 2 dollar 59 cents, do You like to add this to Your list?”

**User:** “Yes.”

**Iris:** “lovely! Added half a liter of Campina fat free milk to the grocery list.” This product is discounted today, should I order it directly?

**User:** “No, thanks”

**Iris:** “Okay, I only added this product to Your list. enjoy Your day!”

Activity #4: (Relational exchanges)

**You want to purchase the grocery list while using Iris.**

**User:** “Iris, which items are on my grocery list?”

**Iris**: “You have four items on Your grocery list:

- Half a liter of Campina fat free milk for 2 dollar and 59 cents.
- 2 rolls of Nalys kitchen paper for 3 dollar and 85 cents.
- 1L of Bertolli olive oil for 9 dollar and 39 cents.
- 23 sun dishwasher cubes for 4 dollar and 79 cents.

The total of all products is 20 dollar 62 cents. Would You like to buy this list?”

**User:** “Yes”

**Iris:** ‘Thank You for Your confirmation. Order request confirmed, please scan Your face to authorize Your purchase.’

**User:** *Scans *

**Iris**: “Thank You, the face identification for this order request is confirmed. Ordered all products from the list. The groceries will be delivered on Friday at 1’o clock in the afternoon.”

Activity #5: (Relational exchanges)

**You want to transfer some money between banking accounts while using Iris.**

**User:** “Iris, check the balance of my checking account.”

**Iris**: “Your checking account available balance is: 100 dollar and 56 cents.”

**User:** “Iris, ask Bank of America to transfer 45 dollar form my checking account to my savings account.”

**Iris**: “You are about the execute a transfer transaction. Please scan Your face to authorize this transfer.

**User:** *Scans *

**Iris**: “Thank You, the face identification for this money transfer is confirmed. Please, say Your PIN-code to perform this transaction.”

**User:** “452957”

**Iris**: “Thank You for Your input. The given PIN correct, your transfer from checking account to savings account of 45 dollar has been successful.”

Activity #6: (Relational exchanges)

**You want to perform a banking transaction while using Iris.**

**User:** “Iris, check the balance of my checking account.”

**Iris**: “Your checking account available balance is: 55 dollar and 56 cents.”

**User:** “Iris, ask Bank of America to send 50 dollar to Peter White.”

**Iris:** “You are about to execute a transaction. Please scan Your face to authorize this transfer.

**User:** *Scans *

**Iris**: “Thank You, the face identification for this transaction is confirmed. Please, say Your PIN-code to perform this transaction.”

**User:** “452957”

**Iris**: “Thank You for Your input. The given PIN correct. I Found Peter White in the contact list. Your transfer from checking account to Peter White of 50 dollar has been successful.”

**Appendix II. Measurement items for each variable in the research model**

| **Variable** | **Items** | **Source** |
| --- | --- | --- |
| Perceived ease of use (PEU) | Interacting with Iris seems like a convenient way to manage my time. | Taylor and Todd (1995) |
|  | Iris seems easy to use. | Davis et al. (1992) |
|  | Completing tasks with Iris would make my life easier. | Taylor and Todd (1995) |
|  | Iris’ her voice sounded easygoing. | Lewis & Hardzinski (2015) |
|  | Iris would make me feel relaxed during the interaction. | Polkosky & Lewis (2013) |
| Perceived usefulness (PU) | Completing tasks with Iris seems like an efficient use of my time. | Taylor and Todd (1995) |
|  | Using Iris would increase my productivity. | Davis et al, (1992) |
|  | Iris seems like a useful interaction system. | Lewis & Hardzinski (2015) |
|  | The quality of the output I would receive from Iris seems high. | Kuo et al .(2017) |
|  | The answers given by Iris are informative. | Waytz et al. (2004) |
| Perceived privacy concerns (PPC) | I would be concerned that my personal details stored by Iris could be stolen. | Mclean & Osei-Frimpong (2019) |
|  | I have doubts over the confidentiality of my interactions with voice technology. | Mclean & Osei-Frimpong (2019) |
|  | I would be concerned to perform a financial transaction via Iris. | Mclean & Osei-Frimpong (2019) |
|  | Iris sounded capable of keeping my personal data safe. | Lewis & Hardzinski (2015) |
|  | I am skeptical towards Iris’ measures to protect my privacy | Reneau (2013). |
| Perceived security risks (PSR) | Iris seems like a safe technology to interact with. | Tayler & Todd (1995) |
|  | I am concerned that Iris collects too much information about me. | Mclean & Osei-Frimpong (2019) |
|  | I would feel confident during the interaction with Iris. | Lee et al (2016) |
|  | Iris sounded like a reliable person. | Polkosky & Lewis (2013) |
|  | I would feel in control during the interaction with Iris. | Lee et al (2006) |
| Adoption of voice intelligence (AVI) | I would use Iris right now | Lewis & Hardzinski (2015) |
|  | It would be likely that I will use Iris more than once. | Lewis & Hardzinski (2015 |
|  | I plan to use voice assistants like Iris in the future. | Venkatesh et al. (2012) |
|  | I predict that I would use voice technology for different activities. | Venkatesh et al. (2012) |
|  | Would you agree to use Iris as an alternative to other smart technologies with touch elements? | Reneau (2013) |
